# Synthesis of d- and l-Phenylalanine Derivatives by Phenylalanine Ammonia Lyases: A Multienzymatic Cascade Process**

**DOI:** 10.1002/anie.201410670

**Published:** 2015-02-26

**Authors:** Fabio Parmeggiani, Sarah L Lovelock, Nicholas J Weise, Syed T Ahmed, Nicholas J Turner

**Affiliations:** Manchester Institute of Biotechnology and School of Chemistry, University of Manchester131 Princess Street, M1 7DN, Manchester (UK)

**Keywords:** amino acids, biocatalysis, enzymes, lyases, synthetic methods

## Abstract

The synthesis of substituted d-phenylalanines in high yield and excellent optical purity, starting from inexpensive cinnamic acids, has been achieved with a novel one-pot approach by coupling phenylalanine ammonia lyase (PAL) amination with a chemoenzymatic deracemization (based on stereoselective oxidation and nonselective reduction). A simple high-throughput solid-phase screening method has also been developed to identify PALs with higher rates of formation of non-natural d-phenylalanines. The best variants were exploited in the chemoenzymatic cascade, thus increasing the yield and ee value of the d-configured product. Furthermore, the system was extended to the preparation of those l-phenylalanines which are obtained with a low *ee* value using PAL amination.

The asymmetric hydroamination of activated alkenes, catalyzed by ammonia lyases, represents a very attractive approach to the synthesis of enantiomerically enriched chiral amino acids. In particular, phenylalanine ammonia lyases (PALs, EC 4.3.1.24), identified in various organisms, mainly plants and yeasts, catalyze the interconversion of cinnamic acids and l-phenylalanines.[1] The reaction is synthetically very valuable since it does not require expensive cofactors or recycling systems, and its practical applications have already been demonstrated on small and large scale.[2]

However, the use of PALs for the production of non-natural d-phenylalanines would increase their value further, since the latter are important building blocks for many natural products (e.g. macrolide antibiotics) and active pharmaceutical ingredients [e.g. nateglinide and PPACK (d-phenylalanyl-l-prolyl-l-arginine chloromethyl ketone)], either as key chiral structural fragments, or in peptides to confer resistance to enzymatic hydrolysis. The PAL-mediated synthesis of d-phenylalanines would represent an atom-economic and low-cost approach compared to alternative methods.[3]

Nonetheless, to date no evidence of wild-type PAL enzymes which are able to produce d-phenylalanines with high selectivity have been found. We recently reported[4] that PAL-catalyzed amination of the cinnamic acids **1** can lead to the formation of significant levels of the enantiomers d-**2** together with the expected l-**2** products (Scheme 1, left). In particular, for cinnamic acids with an electron-deficient aromatic ring, a considerable reduction in the *ee* value of l-**2** was observed over time. Herein, we exploit this activity and describe a system for the synthesis of optically enriched d-amino acids. For this purpose we designed a cascade process involving the deracemization of **2** (i.e., an enantioselective conversion of l-**2** into a prochiral intermediate, along with a nonspecific complementary reaction which regenerates *rac*-**2**, thus resulting in the accumulation of d-**2**).[5] Specifically, we selected a system involving the enzymatic oxidation of l-**2** into the corresponding imino acid **3** and the nonselective chemical reduction of the latter (Scheme 1, right). Such a reduction can be carried out with several reagents, for example, borohydrides, borane complexes, or catalytic transfer hydrogenation. The borane–ammonia complex is the mildest, cheapest, and most efficient option.[6] Its compatibility with proteins and its stability in water at high pH values and high ammonia concentrations made it ideal for our purpose. For the oxidation step, the first choice would be an l-amino acid oxidase (LAAO), although no enzyme of this class with broad substrate specificity has been successfully expressed in a prokaryotic host in an active form.[7] However, the same reaction is also performed by l-amino acid deaminases (LAADs),[8] which are membrane-bound proteins associated with the respiratory electron transport chain, and use oxygen as a co-substrate but produce water instead of H_2_O_2_. We selected the LAAD from the enterobacterium *Proteus mirabilis*,[9] which has been successfully expressed in *E. coli*, is active on a broad spectrum of substrates, and has already been exploited as a whole-cell biocatalyst on preparative scale.[10]

**Scheme 1:**
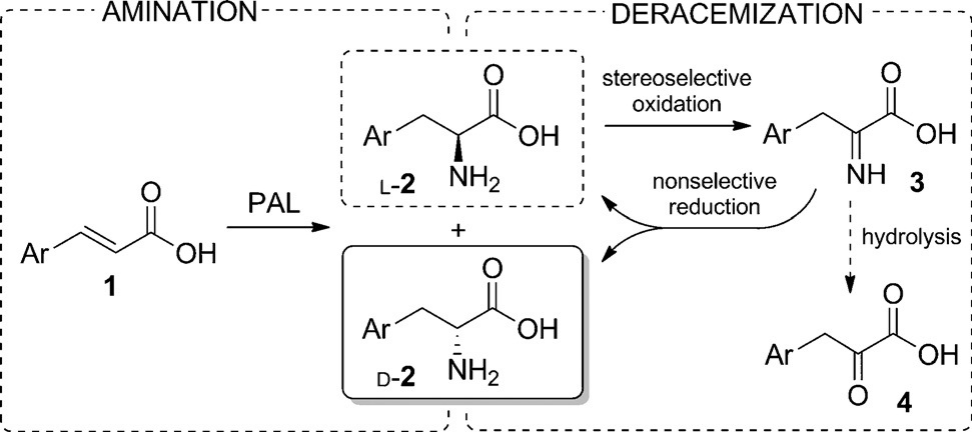
Amination/deracemization cascade concept.

The activity of LAAD in the deracemization of the model substrate *p*-nitrophenylalanine (*rac*-**2 a**) was tested under different conditions (Table 1), at high pH and in the presence of high concentrations of NH_3_, thus confirming its compatibility with the optimal conditions for PAL aminations. The addition of at least 40 equivalents of borane allowed complete deracemization under these conditions. The formation of small amounts of the keto acid **4** (see Scheme 1) by spontaneous hydrolysis of **3** led to a slight reduction in overall yield. The use of the alternative reducing agents mentioned above did not lead to any improvement in conversion (see Table S1 in the Supporting Information).

**Table 1:** Deracemization of *rac*-2 a into d-2 a with LAAD^[a]^

Buffer system	pH	NH_3_:BH_3_ [equiv]	*ee* [%]^[b]^	2 a [%]^[b]^
KP_i_ 100 mm	7.0	30	>99	84
KP_i_ 100 mm	8.0	30	>99	82
NH_4_OH 100 mm	9.0	30	>99	82
NH_4_OH 1 m	9.0	30	96	80
NH_4_OH 3 m	9.0	30	92	81
NH_4_OH 5 m	9.0	30	93	79
NH_4_OH 5 m	9.6	30	95	78
NH_4_OH 5 m	9.6	50	>99	87

[a] Reaction conditions: 5 mm *rac*-**2 a**, 15 mg mL^−1^ LAAD *E. coli* wet cells, 37 °C, 220 rpm, 7 h. [b] Determined by HPLC analysis.

Then we tested the performance of wild-type PAL in the coupled system. The PAL from the cyanobacteria *Anabaena variabilis*[11] is particularly suited for biocatalysis for three reasons: 1) the expression level is higher than nonbacterial PALs, 2) the enzyme possesses a broader substrate specificity,[2f] and 3) the X-ray crystal structure has been solved with the mobile loops in the active closed conformation, thus facilitating mutagenesis studies.

The coupling of PAL amination and LAAD/NH_3_:BH_3_ deracemization (Scheme 1) resulted in 71 % conversion of *p*-nitrocinnamic acid (**1 a**) into d-**2 a** with 96 % *ee*, and would thus be useful for preparative applications compared to alternative methods. However, perfect enantioselectivity could not be obtained, and we therefore decided to try to improve the performance of the PAL enzyme by directed evolution.

Initially we developed a high-throughput screening method for the identification of PAL variants with increased selectivity for the formation of the compounds d-**2**. The assay (Scheme 2) is based on the detection of hydrogen peroxide formed by the reaction of the d-amino acid with a d-amino acid oxidase (DAAO, from *Trigonopsis variabilis*)[12] coexpressed together with the PAL variant, thus yielding the corresponding imino acid **3**. In the presence of horseradish peroxidase (HRP), H_2_O_2_ is reduced to water, and colorless 3,3'-diaminobenzidine (DAB) is oxidized to a polymeric brown dye which accumulates in the colony.[13] The intensity of the color is proportional to the concentration of peroxide, and related in turn to the amount of d-**2** formed by PAL.

**Scheme 2:**
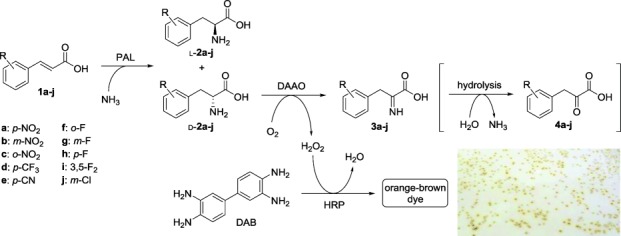
Reactions involved in the solid-phase screening for d-PAL activity.

As a starting pool of variants to test this screening platform, we employed a mixture of 48 separate NNK site-saturation mutagenesis libraries (960 variants) targeting 48 residues in close proximity to the binding site (see Figure S1).[14] The screening was performed as follows (Figure 1): 1) ultracompetent *E. coli* BL21(DE3) cells were transformed with two plasmids, one harbouring the PAL variant and the other the DAAO, and plated onto a nylon membrane laid on an agar plate; 2) colonies were grown overnight at 30 °C; 3) the membrane was transferred to a fresh plate containing IPTG and incubated overnight at 20 °C; 4) partial lysis of the cells was achieved by freeze–thaw cycles in liquid nitrogen; 5) the membrane was then treated with a HRP solution to remove any peroxide present; 6) the membrane was transferred to a filter paper soaked with a solution of substrate, NH_3_, HRP, and DAB, and incubated at room temperature.

**Figure 1:**
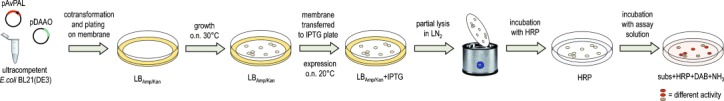
Protocol for the solid-phase screening for d-PAL activity.

*p*-Nitrocinnamic acid (**1 a**) was selected as a model substrate, although similar behavior was observed with other d-forming substrates (**1 b**–**e**): a qualitative estimation of the activity of the variant in each colony could be easily carried out by visual inspection (see inset in Scheme 1 and Figures S2 and S3). No color change was observed when employing the substrates **1 f**–**j**, which are known to afford exclusively the l-enantiomer, even after a prolonged incubation with the wild-type enzyme.[4] The screening was then performed on approximately 5000 colonies and the most-active clones were picked, sequenced, and assayed in analytical-scale biotransformations to measure conversion and product *ee* values by HPLC. All positive clones were found to produce d-**2 a** at a faster or similar rate compared to the wild-type enzyme (see Table S2), and several variants appeared to be considerably faster. To validate the assay, 35 clones randomly picked from the colorless colonies showed low to no formation of d-**2 a** when analysed by HPLC (see Table S3).

To avoid bias resulting from varying levels of protein expression, the most active variants were purified and analytical-scale biotransformations were performed using normalized protein concentrations (see Figure S4). The best mutants thus identified are listed in Table 2. In spite of their enhanced d-PAL activity, none of the variants led to a change in the final position of the equilibrium, thus giving a practically racemic product and no useful enrichment of d-**2 a**, as previously observed with the wild-type enzyme.[4]

**Table 2:** PAL variants with the highest specific d-formation activity^[a]^

Variant	Specific d-PAL activity [U mg^−1^]	Relative activity compared to WT
WT	3.23	–
H359Y	11.35	3.52
H359K	10.76	3.34
S456P	9.83	3.05
A88Q	9.10	2.82
S263A	7.95	2.47

[a] Reaction conditions: 5 mm **1 a**, 5 m NH_4_OH, 25 μg mL^−1^ purified PAL, pH 9.6, 37 °C, 220 rpm, 4 h.

However, we anticipated that these d-PAL variants could be exploited in our amination/deracemization cascade system. By employing the best mutant identified from the screening (H359Y) the *ee* value of d-**2 a** increased to greater than 99 % and the conversion to 78 % under the same reaction conditions, thus further demonstrating the faster production of d-**2 a** with respect to wild-type PAL (other variants behaved similarly; see Table S4).

The amination/deracemization cascade (Scheme 3 a) was then tested with a range of electron-deficient cinnamic acids (**1 b**–**e**), thus affording d-**2 b**–**e** in good yields and excellent optical purities (Table 3). Since the deracemization also works efficiently when starting from pure l-**2**, the same approach was extended successfully to the cinnamic acid substrates **1 f**–**j**, thus leading to optically pure d-**2 f**–**j**. A representative reaction profile for **1 f** is shown in Figure 2. Even with the cascade system, some of the cinnamic acid remained unconverted as a consequence of the reversible nature of the PAL reaction. However, the amount of unconverted **1** is lower than in the case of PAL alone, thus showing at least a partial beneficial effect of the cascade coupling of amination and deracemization (Figure 2 and Table 3).

**Figure 2:**
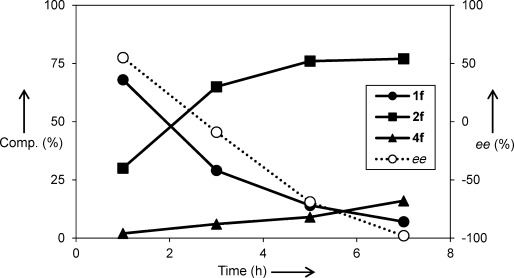
Composition profile and *ee* values for the conversion of 1 f into d-2 f (*ee* values are positive for l-2 f and negative for d-2 f).

**Scheme 3:**
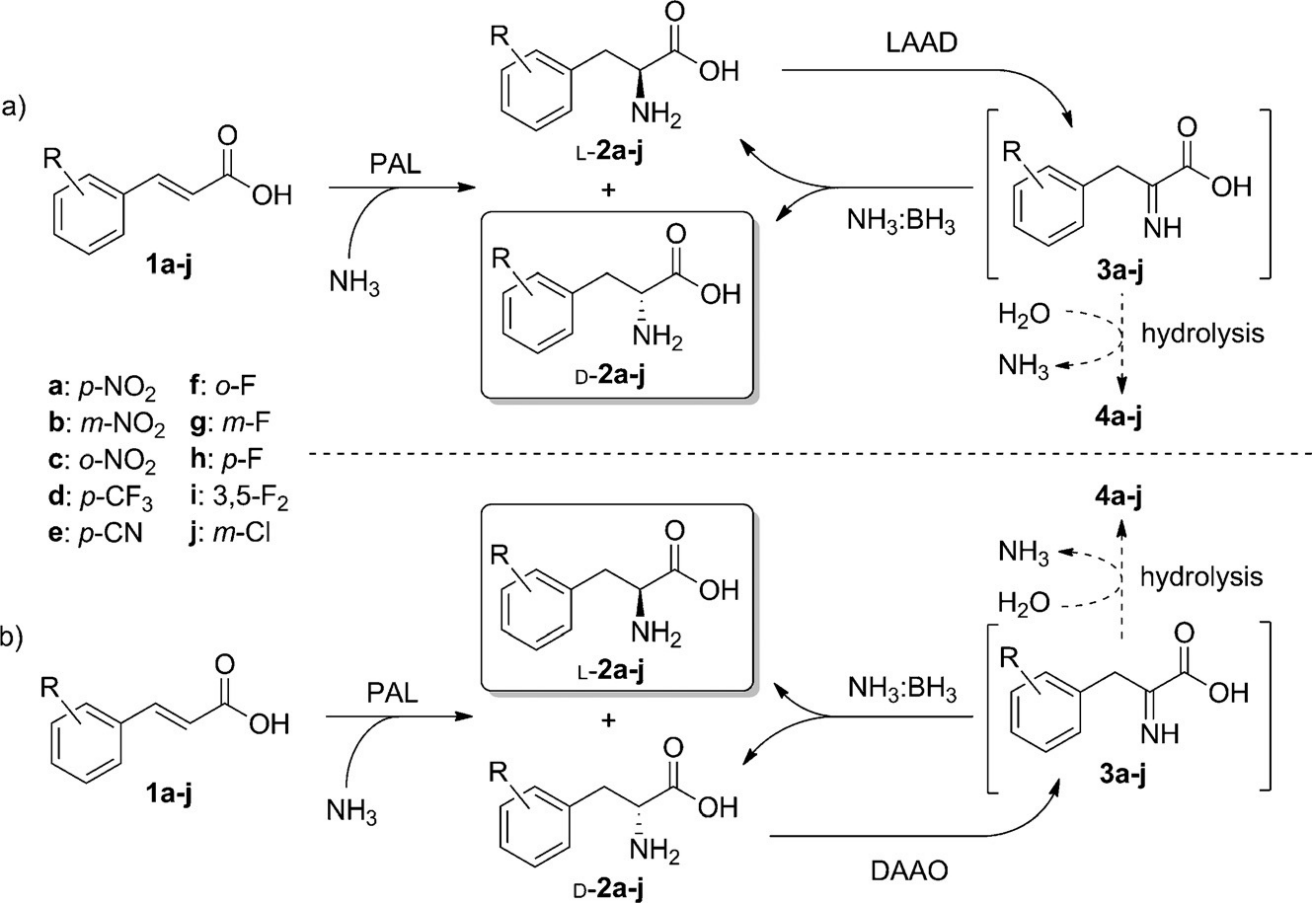
Enantiocomplementary amination/deracemization cascades to afford either optically pure d- or l-2.

**Table 3:** Synthesis of d- and l-2 a–j from 1 a–j^[a]^

		PAL(H359Y)/LAAD cascade		PAL(WT)/DAAO cascade		PAL(WT) only	
Subs.	R	Conv. [%]^[b]^	*ee* d-2[%]^[b]^	Conv. [%]^[b]^	*ee* l-2[%]^[b]^	Conv. [%]^[b]^	*ee* l-2[%]^[b]^
**1 a**	*p*-NO_2_	79	>99	82	>99	84	*rac*
**1 b**	*m*-NO_2_	74	98	76	>99	79	89
**1 c**	*o*-NO_2_	80	98	83	>99	91	*rac*
**1 d**	*p*-CF_3_	64	98	66	>99	65	76
**1 e**	*p*-CN	77	99	79	>99	72	70
**1 f**	*o*-F	77	>99	n.r.	n.r.	84	>99
**1 g**	*m*-F	74	98	n.r.	n.r.	67	>99
**1 h**	*p*-F	62	>99	n.r.	n.r.	53	>99
**1 i**	3,5-F_2_	78	>99	n.r.	n.r.	57	97
**1 j**	*m*-Cl	63	99	n.r.	n.r.	86	>99

[a] Reaction conditions: 5 mm subs., 5 m NH_4_OH, 25 mg mL^−1^ PAL *E. coli* wet cells, 35 mg mL^−1^ LAAD or DAAO *E. coli* wet cells, 40 equiv NH_3_:BH_3_, pH 9.6, 37 °C, 220 rpm. [b] Determined by HPLC analysis (yields of isolated products are given in the Supporting Information). n.r.=not required, because of the high *ee* value products from the PAL-mediated reaction alone.

Furthermore, we were able to adapt this strategy (Scheme 3 b) to the formation of optically pure l-**2 a**–**e** from **1 a**–**e** by employing *E. coli* producing DAAO instead of LAAD. This approach achieves enantiocomplementarity with the same panel of biocatalysts, thus providing access to substituted l-phenylalanines which result in low *ee* values in direct PAL aminations (Table 3).

In conclusion, an efficient high-throughput solid-phase screening assay was successfully developed and used to identify variants with enhanced activity towards d-phenylalanines, thus providing a starting platform for further directed evolution studies. The best variant was exploited in a cascade process to convert a range of cinnamic acids into optically pure d-phenylalanine derivatives. The cascade was also adapted to enhance the enantioselectivity for l-phenylalanines starting from the same cinnamic acids. This approach (employed in both the screening and the preparative application), represents a useful multienzymatic one-pot system involving PALs and thus widens the range of applications of these valuable biocatalysts in preparative biotransformations.

## Supporting Information

As a service to our authors and readers, this journal provides supporting information supplied by the authors. Such materials are peer reviewed and may be re-organized for online delivery, but are not copy-edited or typeset. Technical support issues arising from supporting information (other than missing files) should be addressed to the authors.

miscellaneous_information
