# Supplementary material for: Synthesis of d- and l-Phenylalanine Derivatives by Phenylalanine Ammonia Lyases: A Multienzymatic Cascade Process
Source: Angew Chem Int Ed Engl. 2015 Feb 26;54(15):4608–11. doi: 10.1002/anie.201410670 (PMC4531825; doi:10.1002/anie.201410670)
Supplement: Supplementary file 1 — miscellaneous_information [file anie0054-4608-sd1.pdf]

Angewandte Chemie
Eine Zeitschrift der Gesellschaft Deutscher Chemiker

Supporting Information

# Synthesis of D- and L-Phenylalanine Derivatives by Phenylalanine Ammonia Lyases: A Multienzymatic Cascade Process**

*Fabio Parmeggiani, Sarah L. Lovelock, Nicholas J. Weise, Syed T. Ahmed, and Nicholas J. Turner**

anie_201410670_sm_miscellaneous_information.pdf

# SUPPORTING INFORMATION

**Table of contents** **Page**

Figures S1-S4 ........ S2
Tables S1-S4 ........ S5
Experimental section ........ S8
Chiral HPLC chromatograms of compounds **2a-j** ........ S13
NMR spectra of compounds **2a-j** ........ S17
References ........ S22

**Figures S1-S4**

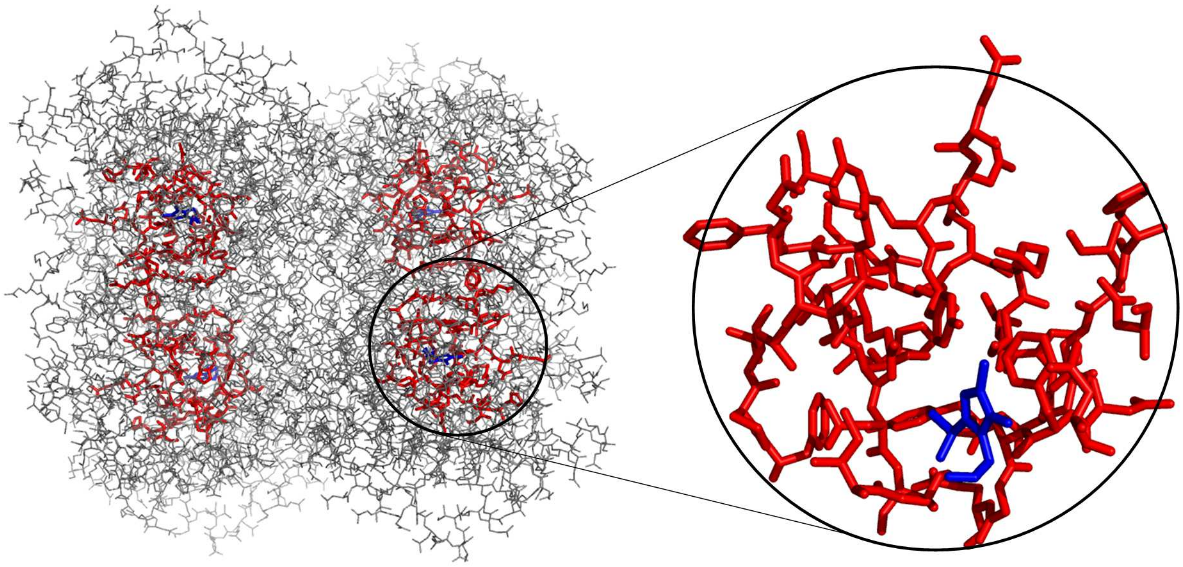

| | | | | | | | |
|---|---|---|---|---|---|---|---|
| Y78 | S168 | R313 | L319 | G360 | Q366 | P444 | N451 |
| G85 | N223 | Y314 | P320 | G361 | H385 | T445 | Q452 |
| G86 | S263 | S315 | Q321 | N362 | V388 | A447 | N453 |
| A88 | L309 | L316 | Y322 | F363 | Q389 | E448 | I454 |
| I165 | Q311 | R317 | N347 | L364 | L392 | Q449 | N455 |
| G166 | D312 | C318 | H359 | G365 | F443 | F450 | S456 |

**Figure S1.** Library of single-point AvPAL mutants employed as a test bed for the solid phase screening. The codons corresponding to the residues shown in red and listed, have been replaced with NNK (combination of 48 separate NNK libraries). The catalytic MIO cofactor is shown in blue.

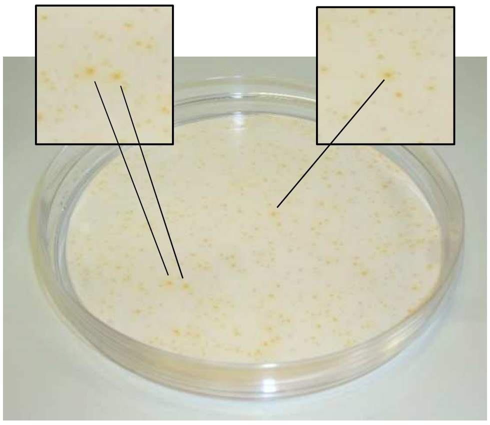

**Figure S2.** High-throughput solid-phase screening for D-PAL activity on substrate **1h**. The insets show highly active colonies.

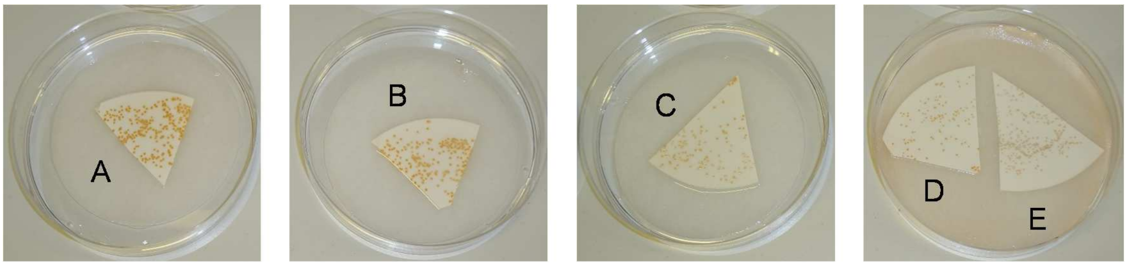


**Figure S3.** Control experiments for the solid-phase screening performed on portions of membranes. A-C: cells transformed with pET16b-wtAvPAL, the assay mix contains D-**2h** at different concentrations instead of **1h** (A: 5 mM, B: 1 mM, C: 0.2 mM), showing that the intensity of the colour is correlated to the amount of D-product present. D-E: assay with substrate **1h** (D: cells transformed with pET16b-wtAvPAL, E: cells transformed with empty pET16b), showing that the wild type has moderate but detectable activity, while no background activity from *E. coli* can be observed.

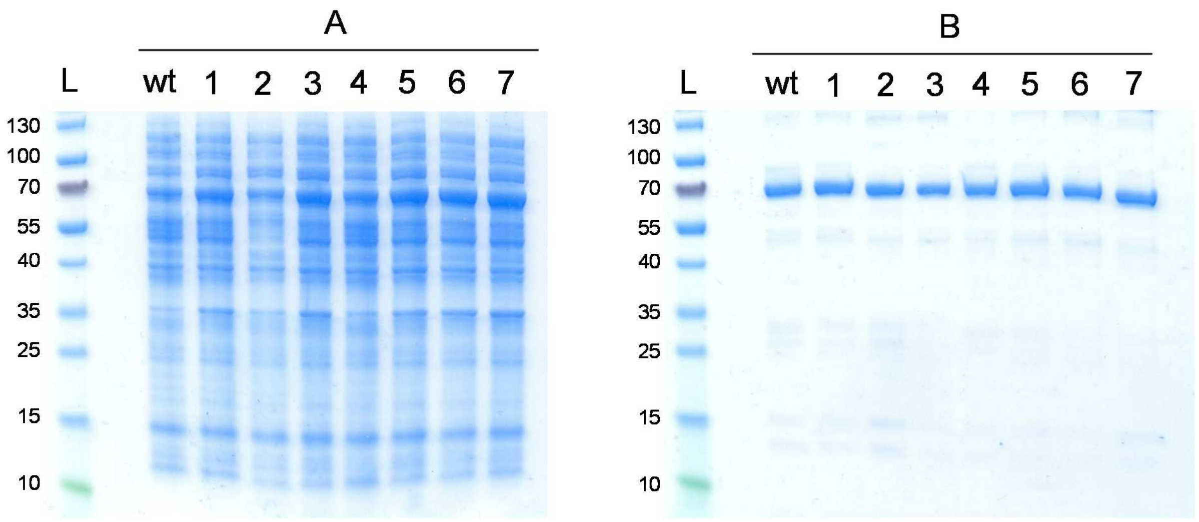


**Figure S4.** SDS-PAGE analysis of AvPAL variants. A: cell-free extracts (showing slightly different expression levels); B: purified protein samples with normalised concentration; L = prestained protein ladder.

## Tables S1-S4

**Table S1.** Deracemisation of *rac*-**2a** to D-**2a** with alternative reducing agents.

| Reducing agent | ee (%)[a] | Yield (%)[a] |
|---|---|---|
| $NH_3$:$BH_3$ | 95 | 78 |
| *t*-Bu$NH_2$:$BH_3$ | 91 | 81 |
| morpholine:$BH_3$ | 8 | 77 |
| $Me_3N$:$BH_3$ | 7 | 97 |
| $NaBH_3CN$ | 9 | 91 |
| $HCOONH_4$, Pd/C (cat.)[b] | –[c] | –[c] |

Expt. cond.: 5 mM *rac*-**2a**, $NH_4OH$ 5 M, 15 mg mL$^{-1}$ LAAD *E. coli* wet cells, 30 eq. reducing agent, pH 9.6, 37°C, 220 rpm, 7 h. a: by HPLC; b: $HCOONH_4$ 5 M has been used in place of $NH_4OH$ 5 M; c: side reactions were observed.

$NaBH_3CN$ and borane complexes with secondary or tertiary amines did not perform well, while with catalytic transfer hydrogenation ($HCOONH_4$, Pd/C), side reactions such as reduction of the nitro group (and of the C=C bond of cinnamic acid in the case of cascade reactions) were observed. Instead, *t*-Bu$NH_2$:$BH_3$ was found as effective as $NH_3$:$BH_3$, but the latter is more convenient because of its lower cost and higher atom-economy.

**Table S2.** AvPAL variants with D-formation activity higher or similar to the wild type (positive hits from the solid-phase screening, verified by HPLC analysis).

| | | | | |
|---|---|---|---|---|
| F450C | H359D | V388G | Q366H | H359A |
| V388T | S315P | H359L | F450G | S263G |
| F450A | L319V | F450G | H359Q | V388M |
| Y322A | S456P | F450M | V388Q | H359C |
| P444C | R313G | Q449D | F450L | S263N |
| L364H | Q366S | T445I | L392H | L364M |
| L392N | F450H | H359E | Q366E | L392F |
| S315G | N453G | Q366P | N453H | N453A |
| H385G | S263C | L392M | I454T | L392T |
| N453A | H385C | P444A | Q321H | H385Q |
| F443A | I454L | H385M | S263N | N453G |
| F450S | Q389G | V388I | N347T | L392Q |

**Table S3.** AvPAL variants with no D-formation activity (negative hits from the solid-phase screening, verified by HPLC analysis).

| | | | | |
|---|---|---|---|---|
| F443R | Q321E | Q452L | Y78F | N451R |
| Y78H | I454Y | I165R | F363R | S456R |
| T445R | F363P | Y78D | N362E | A88R |
| G361V | Q452L | Q449R | Y78A | L392P |
| F443K | S263R | Q452K | N223Y | G365D |
| Q452N | Y78I | R317L | Q389R | Q389P |
| G85R | N347C | N362R | F443H | Y78P |

**Table S4.** Cascade process for the synthesis of D-**2a** from **1a** with selected AvPAL mutants.

| AvPAL mutant | Yield (%) | ee (%) |
|---|---|---|
| wt | 71 | 96 |
| H359Y | 78 | >99 |
| H359K | 73 | >99 |
| S456P | 72 | 97 |

Expt. cond.: 5 mM **1a**, 5 M $NH_4OH$, 25 mg $mL^{-1}$ PAL *E. coli* wet cells, 35 mg $mL^{-1}$ LAAD *E. coli* wet cells, 40 eq. $NH_3$:$BH_3$, pH 9.6, 37°C, 220 rpm, 8 h.

## Experimental section

### General methods

Analytical grade reagents were obtained from Sigma-Aldrich, AlfaAesar or Fisher Scientific and used as received from the supplier.

Reverse phase HPLC was performed on an Agilent system equipped with a G1379A degasser, G1312A binary pump, a G1329 autosampler unit, a G1316A temperature controlled column compartment and a G1315B diode array detector.

Conversions were monitored by reverse phase HPLC on a Zorbax Extend-C18 column (50 mm × 4.6 mm × 3.5 μm, Agilent). For compounds **1a-c** and **1e-h**: mobile phase aq. $NH_4OH$ 0.1 M pH 10 / MeOH 9:1, flow rate 1.0 mL $min^{-1}$, temperature 40°C, detection wavelength 210 nm. Retention times: **1a** 5.59 min, **2a** 2.68 min, **1b** 6.16 min, **2b** 2.67 min, **1c** 5.55 min, **2c** 2.78 min, **1e** 3.74 min, **2e** 1.94 min, **1f** 6.54 min, **2f** 2.61 min, **1g** 6.98 min, **2g** 2.77 min, **1h** 6.45 min, **2h** 2.69 min. For compound **1i** mobile phase aq. $NH_4OH$ 0.1 M pH 10 / MeOH 8:2, flow rate 1.0 mL $min^{-1}$, temperature 40°C, detection wavelength 210 nm. Retention times: **1i** 5.57 min, **2i** 2.21 min. For compounds **1d** and **1j**: mobile phase aq. $NH_4OH$ 0.1 M pH 10 / MeOH 7:3, flow rate 1.0 mL $min^{-1}$, temperature 40°C, detection wavelength 210 nm. Retention times: **1d** 8.27 min, **2d** 3.82 min, **1j** 4.65 min, **2j** 2.20 min. The conversions were calculated from the peak areas using standard calibration curves.

Amino acids ee values were determined by reverse phase HPLC on a Crownpak-CR-(+) column (150 mm × 4 mm × 5 μm, Daicel). For compounds **2a-c** and **2e-i**: mobile phase aq. $HClO_4$ 1.14% w/v / MeOH 96:4, flow rate 1.0 mL $min^{-1}$, temperature 40°C, detection wavelength 210 nm. Retention times: D-**2a** 7.52 min, L-**2a** 8.71 min, D-**2b** 7.40 min, L-**2b** 10.63 min, D-**2c** 6.54 min, L-**2c** 8.14 min, D-**2e** 4.69 min, L-**2e** 5.53 min, D-**2f** 5.84 min, L-**2f** 7.51 min, D-**2g** 6.47 min, L-**2g** 8.34 min, D-**2h** 6.82 min, L-**2h** 8.19 min, D-**2i** 7.36 min, L-**2i** 9.03 min. For compounds **2d** and **2j**: mobile phase aq. $HClO_4$ 1.14% w/v / MeOH 86:14, flow rate 1.0 mL $min^{-1}$, temperature 40°C, detection wavelength 210 nm. Retention times: D-**2d** 20.37 min, L-**2d** L 25.19 min, D-**2j** 11.14 min, L-**2j** 15.23 min.

$^1H$ NMR spectra were recorded on a Bruker Avance 400 (400.1 MHz) without additional internal standard. Chemical shifts are reported as δ in parts per million (ppm) and are calibrated against residual solvent signal.

Plasmid DNA purification, digestion and ligation were performed with commercial kits or enzymes (New England Biolabs or Qiagen), according to the manufacturer's instructions. Automated DNA sequencing of the AvPAL gene was performed by MWG Eurofins, using the following primers:

AvPAL_seqFw 5'-ATACCGATATTCTGCAGGG-3',

AvPAL_seqRv 5'-AGATCAACTGCCTGAACAC-3'.

Protein concentrations were measured with the Bradford assay, using bovine serum albumin (BSA) as a standard.

### Strains and plasmids

*E. coli* DH5α was used as a cloning host for plasmid propagation, *E. coli* BL21(DE3) as an expression host for protein production.

The pET16b-AvPAL plasmid containing the *pal* gene from *Anabaena variabilis*, codon-optimised for *E. coli*, was obtained as described previously.[1] This plasmid was used as a starting template for the production of the AvPAL single-point mutants library (provided by Codexis, Inc.).
The pRSF-DAAO plasmid containing the *daao* gene from *Trigonopsis variabilis* was obtained as described previously.[2]
The *pma* gene from *Proteus mirabilis*, codon-optimised for *E. coli*, was synthesised by Geneart and cloned in pET-28a (Novagen) at the restriction sites *Nde*I and *Hin*dIII. The resulting pET28a-LAAD plasmid, checked by sequencing, was used to transform competent *E. coli* cells.
Antibiotics were added where appropriate to the following final concentrations: ampicillin (Amp) 100 µg $mL^{-1}$, kanamycin (Kan) 60 µg $mL^{-1}$. Solid media were prepared by addition of agar (1.5% w/v) to liquid media.
Transformations of chemically competent cells were performed by incubating plasmid DNA (25-50 ng) with the cells (1 aliquot) for 30 min on ice before heat shock (42°C, 20 s). SOC medium (1 mL) was immediately added, and the cells were incubated for 1 h at 37°C and 220 rpm. Colonies containing recombinant plasmids were selected on LB-agar plates containing the suitable antibiotic.

**Solid phase screening of AvPAL variants**

Ultracompetent E.coli BL21-Gold (DE3) cells (1 aliquot, 100 µL, Agilent) were co-transformed with the pET16b-AvPAL library (200 ng) and pRSF-DAAO (100 ng), and plated onto two nylon membranes (Amersham Nybond-N, 132 mm diameter, GE Healthcare) laid on LB-agar plates containing ampicillin and kanamycin. The plates were incubated at 30°C overnight, then the membranes were transferred onto fresh LB-agar plates containing ampicillin, kanamycin and IPTG (1 mM), and incubated at 20°C overnight. The membranes were removed from the plates and dipped three times in liquid nitrogen for 1-2 s, to achieve partial lysis. Then they were placed on filter paper soaked with a solution of HRP (1 mg $mL^{-1}$ in $KP_i$ buffer 100 mM, pH 8.0) and incubated at r. t. for 1 h. The assay solution was prepared adding HRP solution (300 µL, 1 mg $mL^{-1}$ in $KP_i$ buffer 100 mM, pH 8.0) and DAB (1.2 mL, 2 SigmaFast tablets, Sigma-Aldrich, dissolved in 2 mL $KP_i$ buffer 100 mM, pH 8.0 + 2 mL $NH_4OH$ 5 M pH 9.6 in an ultrasonic bath and filtered) to a solution of the substrate (1.5 mL, 20 mM in $NH_4OH$ 5 M pH 9.6). Quantities are given per membrane. The assay was performed by transferring the membranes to a Petri dish containing a filter paper disk soaked in the assay solution and incubating at room temperature until the colour had developed. Clones were picked with sterile toothpicks and transferred to 96-deep-well plates containing 1 mL of LB medium with ampicillin, and grown overnight at 37°C and 220 rpm.

**Expression of AvPAL variants**

A single colony of *E. coli* BL21(DE3) carrying the pET16b-AvPAL plasmid (with the desired mutation) was used to inoculate LB medium (8 mL) supplemented with ampicillin, which was grown overnight at 37°C and 220 rpm. The starter culture was used to inoculate LB-based autoinduction medium[3] (800 mL, ForMedium™) containing ampicillin and glycerol (0.5% v/v) which was incubated at 18°C and 250 rpm for 4 days. The cells were harvested by centrifugation (5,000 rpm, 20 min, 4°C), washed with $KP_i$

buffer (100 mM, pH 8.0) and harvested again by centrifugation (5,000 rpm, 20 min, 4 °C). The cell pellet was aliquoted and stored at –20°C.

**Biotransformations with whole cells containing AvPAL variants**

*E. coli* BL21(DE3) whole cells containing the PAL variant (75 mg wet weight) were resuspended in a solution of substrate (5 mM) in $NH_4OH$ buffer (3 mL, 5 M, pH 9.6). The mixture was incubated at 37°C and 220 rpm. Biotransformation samples (300 μL) were mixed with MeOH (300 μL), thoroughly shaken and centrifuged (13000 rpm, 1 min). The supernatant was transferred to a filter vial and used directly for HPLC analysis.

**Purification of AvPAL variants**

*E. coli* BL21(DE3) whole cells containing the PAL variant (3.5 g wet weight) were resuspended in wash buffer (15 mL, 50 mM $KP_i$, 500 mM NaCl, 20 mM imidazole, pH 7.4). Lysozyme (500 μL, 10 mg $mL^{-1}$) was added and the mixture was incubated at 37°C and 220 rpm for 45 min. The suspension was sonicated (20 s on, 20 s off, 20 cycles, Soniprep 150, MSE UK Ltd) on ice and treated with DNAse (100 μL, 1 mg $mL^{-1}$) at 37°C and 220 rpm for 45 min. The mixture was centrifuged (18,000 rpm, 30 min, 4°C) and the supernatant was filtered (0.2 μm syringe filter) and loaded onto a pre-packed HisTrap FF column (GE Healthcare, 1 mL solid phase). The column was washed with the same wash buffer (5-10 mL) and the protein was eluted with the elution buffer (10 mL, 50 mM $KP_i$, 500 mM NaCl, 250 mM imidazole, pH 7.4), collecting the eluate in different fractions. Fractions were pooled according to the protein concentration (measured by Bradford assay) and purity (judged by SDS-PAGE analysis). The pooled fractions were either desalted on a disposable PD-10 column (GE Healthcare) or used without further processing. If necessary, the fractions were concentrated on a disposable centrifugal filter (100 kDa MWCO, Sartorius Stedim).

**Biotransformations with purified AvPAL variants**

The purified AvPAL variant (50 μL of a 0.5 mg $mL^{-1}$ solution in $KP_i$ buffer 50 mM, pH 7.4) was added to a solution of the substrate (5 mM, 450 μL) in $NH_4OH$ 5 M, pH 9.6. The mixture was incubated at 37°C, 220 rpm. Samples were treated as described for whole cell biotransformations.

**Production of LAAD and DAAO in *E. coli* BL21(DE3)**

A single colony of *E. coli* BL21(DE3) cells transformed with the pRSF-DAAO or the pET28a-LAAD plasmid was used to inoculate LB medium (8 mL) supplemented with kanamycin, which was grown overnight at 37°C and 220 rpm. The starter culture was used to inoculate LB medium (800 mL) containing kanamycin, which was incubated at 37°C and 160 rpm. When an optical density ($OD_{600}$) of 0.4-0.6 was reached, IPTG was added (final concentration 1 mM) and the culture was incubated at 30°C and 160 rpm for 5 h. The cells were harvested by centrifugation (5,000 rpm, 20 min, 4°C), washed with $KP_i$ buffer (100 mM, pH 8.0) and harvested again by centrifugation (5,000 rpm, 20 min, 4°C). The cell pellet was aliquoted and stored at –20°C.

**Deracemisation mediated by LAAD and borane**

*E. coli* BL21(DE3) whole cells containing LAAD (45-75 mg wet weight) were resuspended in a solution of racemic or L-amino acid (5 mM) in the required buffer (3 mL). The mixture was incubated at 37°C and 220 rpm. Samples were treated as described for whole cell biotransformations.

**Cascade synthesis of D-phenylalanines from cinnamic acids**

Method A. The substrate **1a-j** (15 µmol) and $NH_3$:$BH_3$ (40 eq.) were dissolved in $NH_4OH$ 5 M, pH 9.6 (3 mL). *E. coli* BL21(DE3) whole cells containing AvPAL-H359Y (75 mg wet weight) and *E. coli* BL21(DE3) whole cells containing LAAD (105 mg wet weight) were added to the solution and resuspended. The mixture was incubated at 37°C and 220 rpm. Samples were treated as described for whole cell biotransformations.

**Cascade synthesis of L-phenylalanines from cinnamic acids**

Method B. Only substrates **1a-e** were employed. The same procedure described for D-phenylalanines is followed, using *E. coli* BL21(DE3) whole cells containing AvPAL-wt instead of the variant, and *E. coli* BL21(DE3) whole cells containing DAAO instead of LAAD.

**Preparative scale reactions**

The same procedures described above (Method A and Method B) were followed, using 0.5 mmol (80-110 mg) of substrate in a total volume of 100 mL. After the ee reached the maximum value (checked by HPLC), the solution was acidified slowly with aq. $H_2SO_4$ (20% w/v), centrifuged (4000 g, 10 min, 20°C) and applied to a column containing washed Dowex 50W X 8 resin. The resin was washed with water until the eluate was neutral, then the product was eluted with aq. $NH_4OH$ (10% w/v). The fractions containing the product (as measured spectrophotometrically at 280 nm) were pooled and evaporated in a centrifugal evaporator (Genevac).

*p*-Nitrophenylalanine **2a**

From substrate **1a**: D-**2a** (56 mg, 53% isolated yield, >99% ee by HPLC) was obtained with method A; L-**2a** (57 mg, 57% isolated yield, >99% ee by HPLC) was obtained with method B.

$^1$H NMR ($D_2O$+NaOH): δ 8.06 (d, 2H, J = 8.6, Ar*H*), 7.32 (d, 2H, J = 8.6, Ar*H*), 3.42 (dd, 1H, J = 7.2, 6.1, C*H*$NH_2$), 2.95 (dd, J = 13.6, 6.1, 1H, C*H*H), 2.84 (dd, J = 13.6, 7.2, 1H, CH*H*).

*m*-Nitrophenylalanine **2b**

From substrate **1b**: D-**2b** (55 mg, 53% isolated yield, 98% ee by HPLC) was obtained with method A; L-**2b** (58 mg, 55% isolated yield, >99% ee by HPLC) was obtained with method B.

$^1$H NMR ($D_2O$+NaOH): δ 8.00 (m, 2H, Ar*H*), 7.52 (m, 1H, Ar*H*), 7.43 (m, 1H, Ar*H*), 3.41 (dd, 1H, J = 7.2, 6.0, C*H*$NH_2$), 2.95 (dd, J = 13.6, 6.0, 1H, C*H*H), 2.84 (dd, J = 13.6, 7.2, 1H, CH*H*).

*o*-Nitrophenylalanine **2c**

From substrate **1c**: D-**2c** (54 mg, 51% isolated yield, 98% ee by HPLC) was obtained with method A; L-**2c** (61 mg, 58% isolated yield, >99% ee by HPLC) was obtained with method B.

$^1$H NMR ($D_2O$+NaOH): δ 7.87 (d, 1H, J = 8.0, Ar*H*), 7.52 (m, 1H, Ar*H*), 7.33 (m, 1H, Ar*H*), 3.42 (dd, 1H, J = 8.0, 7.2, C*H*$NH_2$), 3.07 (dd, J = 13.4, 7.2, 1H, C*H*H), 3.00 (dd, J = 13.4, 8.0, 1H, CH*H*).

*p*-Trifluoromethylphenylalanine **2d**

From substrate **1d**: D-**2d** (60 mg, 52% isolated yield, 98% ee by HPLC) was obtained with method A; L-**2d** (66 mg, 57% isolated yield, >99% ee by HPLC) was obtained with method B.

$^1$H NMR ($D_2O$+NaOH): δ 7.55 (d, J = 8.0, 2H, Ar*H*), 7.30 (d, J = 8.0, 2H, Ar*H*), 3.41 (dd, J = 7.6, 6.0, 1H, C*H*$NH_2$), 2.92 (dd, J = 13.4, 6.0, 1H, C*H*H), 2.80 (dd, J = 13.4, 7.6, 1H, CH*H*).

*p*-Cyanophenylalanine **2e**

From substrate **1e**: D-**2e** (59 mg, 62% isolated yield, 99% ee by HPLC) was obtained with method A; L-**2e** (56 mg, 58% isolated yield, >99% ee by HPLC) was obtained with method B.

$^1$H NMR ($D_2O$+NaOH): δ 7.65 (d, 2H, J = 8.2, Ar*H*), 7.34 (d, 2H, J = 8.2, Ar*H*), 3.46 (dd, 1H, J = 7.1, 6.2, C*H*$NH_2$), 2.96 (dd, J = 13.4, 6.2, 1H, C*H*H), 2.86 (dd, J = 13.4, 7.1, 1H, CH*H*).

*o*-Fluorophenylalanine **2f**

From substrate **1f**: D-**2f** (48 mg, 52% isolated yield, >99% ee by HPLC) was obtained with method A.

$^1$H NMR ($D_2O$+NaOH): δ 7.17 (m, 2H, Ar*H*), 7.01 (m, 2H, Ar*H*), 3.40 (m, 1H, C*H*$NH_2$), 2.90 (dd, J = 13.6, 6.0, 1H, C*H*H), 2.77 (dd, J = 13.6, 7.6, 1H, CH*H*).

*m*-Fluorophenylalanine **2g**

From substrate **1g**: D-**2g** (52 mg, 56% isolated yield, 98% ee by HPLC) was obtained with method A.

$^1$H NMR ($D_2O$+NaOH): δ 7.22 (m, 1H, Ar*H*), 6.91 (m, 3H, Ar*H*), 3.38 (dd, 1H, J = 7.2, 6.0, C*H*$NH_2$), 2.86 (dd, J = 13.6, 6.0, 1H, C*H*H), 2.72 (dd, J = 13.6, 7.2, 1H, CH*H*).

*p*-Fluorophenylalanine **2h**

From substrate **1h**: D-**2h** (45 mg, 49% isolated yield, >99% ee by HPLC) was obtained with method A.

$^1$H NMR ($D_2O$+NaOH): δ 7.11 (m, 2H, Ar*H*), 6.95 (m, 2H, Ar*H*), 3.34 (dd, 1H, J = 7.2, 6.0, C*H*$NH_2$), 2.81 (dd, J = 13.6, 6.0, 1H, C*H*H), 2.71 (dd, J = 13.6, 7.2, 1H, CH*H*).

3,5-Difluorophenylalanine **2i**

From substrate **1i**: D-**2i** (56 mg, 55% isolated yield, >99% ee by HPLC) was obtained with method A.

$^1$H NMR ($D_2O$+NaOH): δ 6.72 (m, 3H, Ar*H*), 3.41 (m, 1H, C*H*$NH_2$), 2.86 (dd, J = 13.4, 5.6, 1H, C*H*H), 2.74 (dd, J = 13.4, 7.2, 1H, CH*H*).

*m*-Chlorophenylalanine **2j**

From substrate **1j**: D-**2j** (46 mg, 45% isolated yield, 99% ee by HPLC) was obtained with method A.

$^1$H NMR ($D_2O$+NaOH): δ 7.18 (m, 3H, Ar*H*), 7.06 (m, 1H, Ar*H*), 3.36 (dd, 1H, J = 7.2, 5.6, C*H*$NH_2$), 3.83 (dd, J = 13.3, 5.6, 1H, C*H*H), 2.70 (dd, J = 13.3, 7.2, 1H, CH*H*).

## Chiral HPLC chromatograms of compounds 2a-j

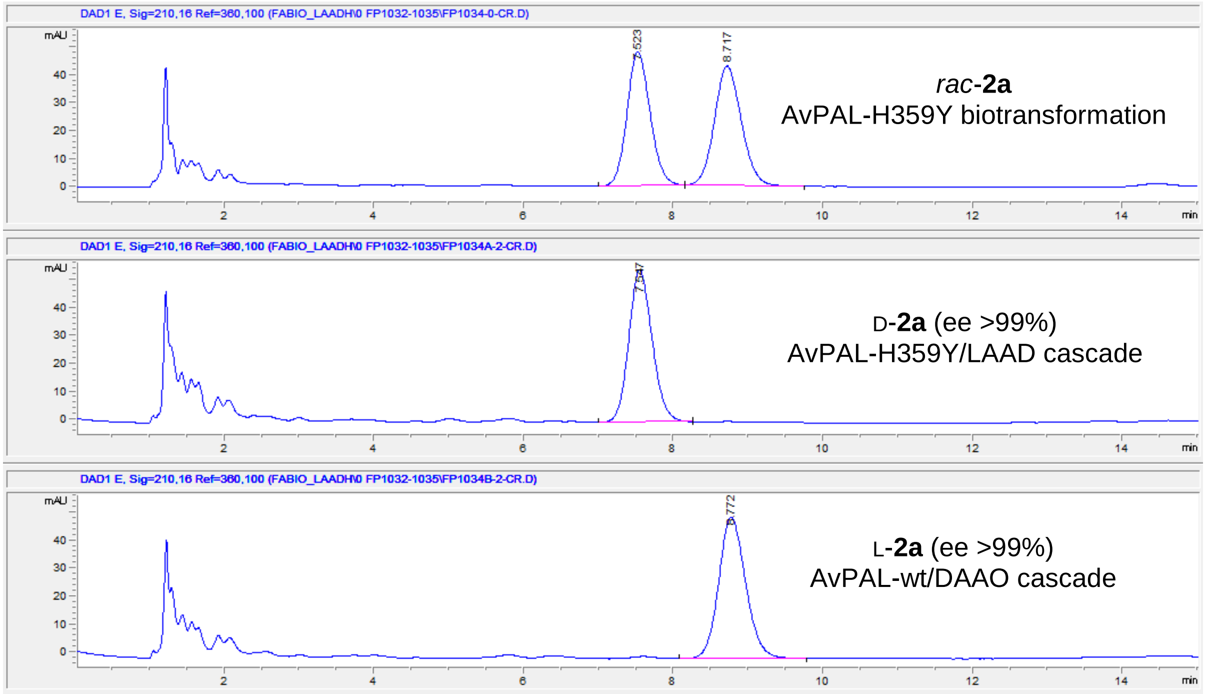


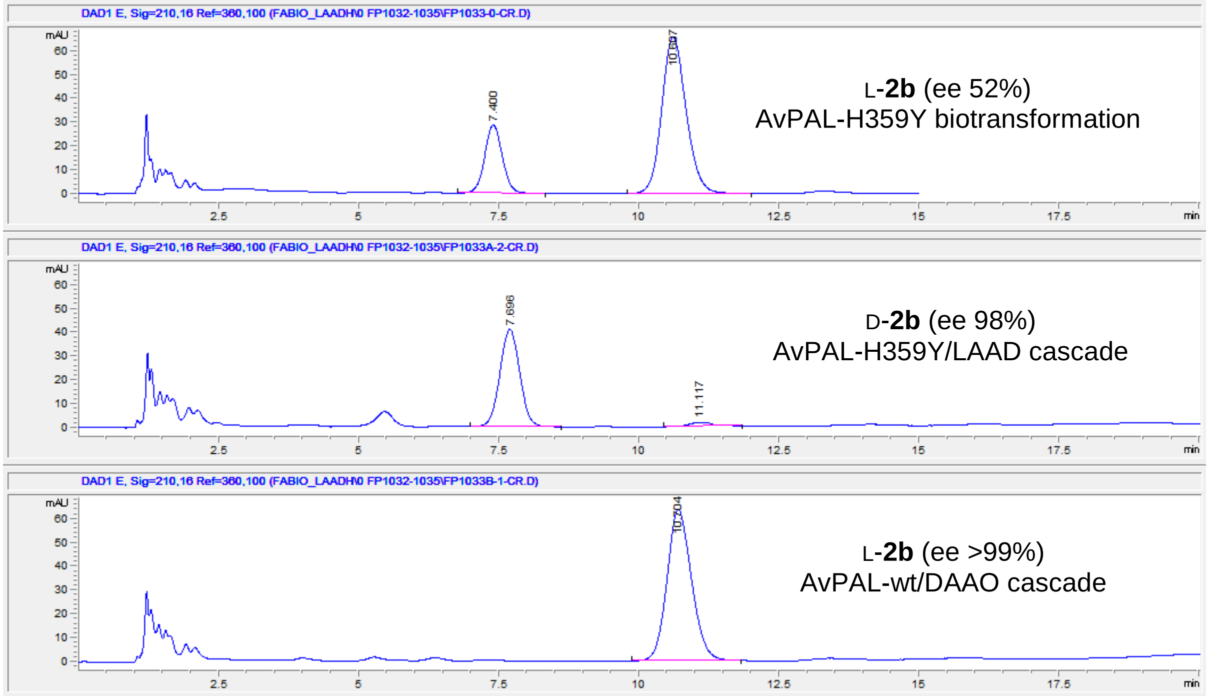

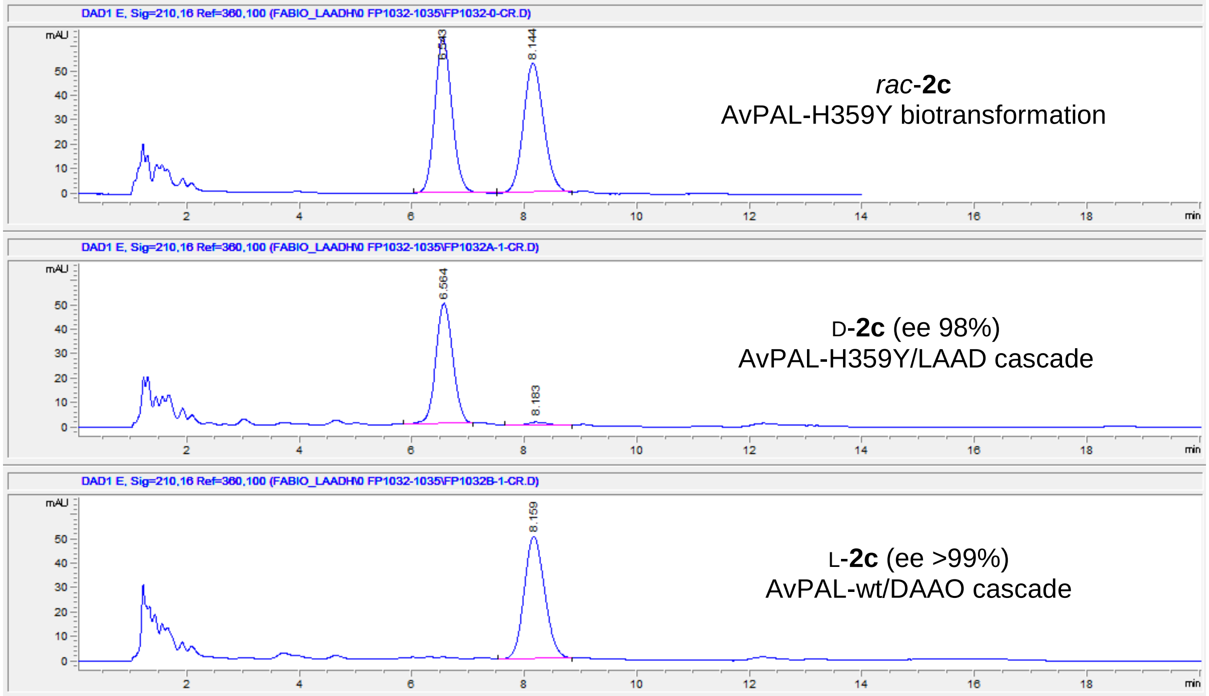
DAD1 E, Sig=210,16 Ref=360,100 (FABIO_LAADH\0 FP1032-1035\FP1032-0-CR.D)
6.543
8.144
rac-2c
AvPAL-H359Y biotransformation
DAD1 E, Sig=210,16 Ref=360,100 (FABIO_LAADH\0 FP1032-1035\FP1032A-1-CR.D)
6.564
8.183
D-2c (ee 98%)
AvPAL-H359Y/LAAD cascade
DAD1 E, Sig=210,16 Ref=360,100 (FABIO_LAADH\0 FP1032-1035\FP1032B-1-CR.D)
8.159
L-2c (ee >99%)
AvPAL-wt/DAAO cascade
mAU
min

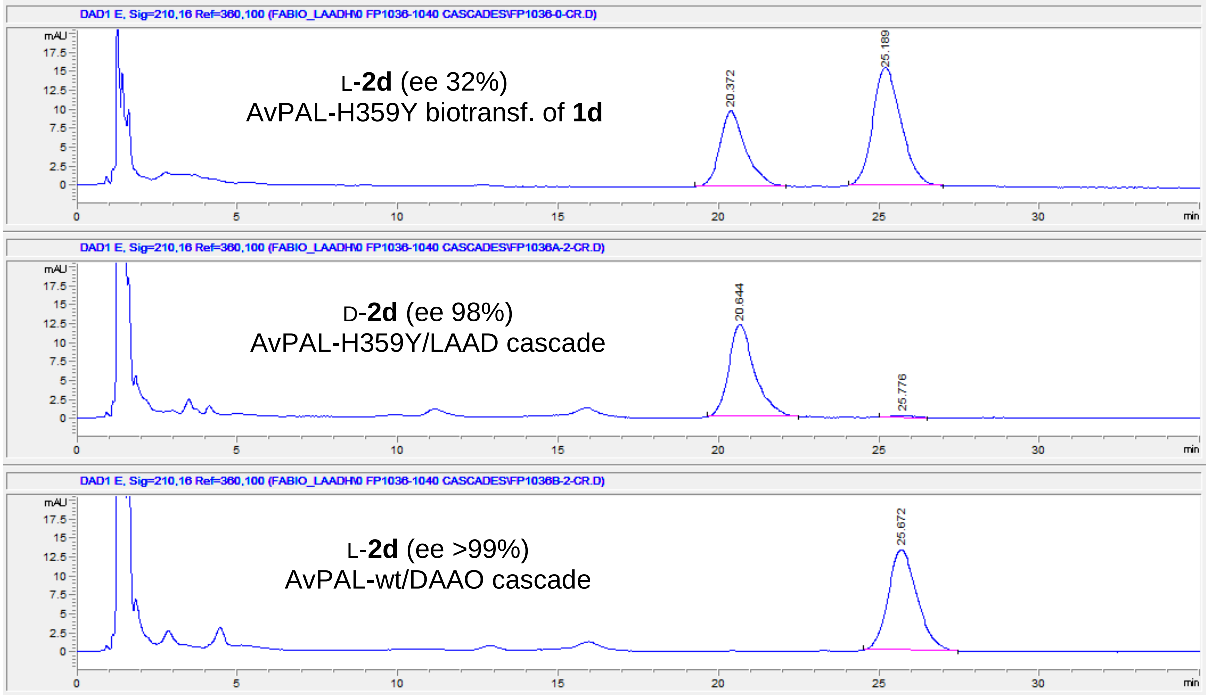
DAD1 E, Sig=210,16 Ref=360,100 (FABIO_LAADH\0 FP1036-1040 CASCADES\FP1036-0-CR.D)
L-2d (ee 32%)
AvPAL-H359Y biotransf. of 1d
20.372
25.189
DAD1 E, Sig=210,16 Ref=360,100 (FABIO_LAADH\0 FP1036-1040 CASCADES\FP1036A-2-CR.D)
D-2d (ee 98%)
AvPAL-H359Y/LAAD cascade
20.644
25.776
DAD1 E, Sig=210,16 Ref=360,100 (FABIO_LAADH\0 FP1036-1040 CASCADES\FP1036B-2-CR.D)
L-2d (ee >99%)
AvPAL-wt/DAAO cascade
25.672
mAU
min

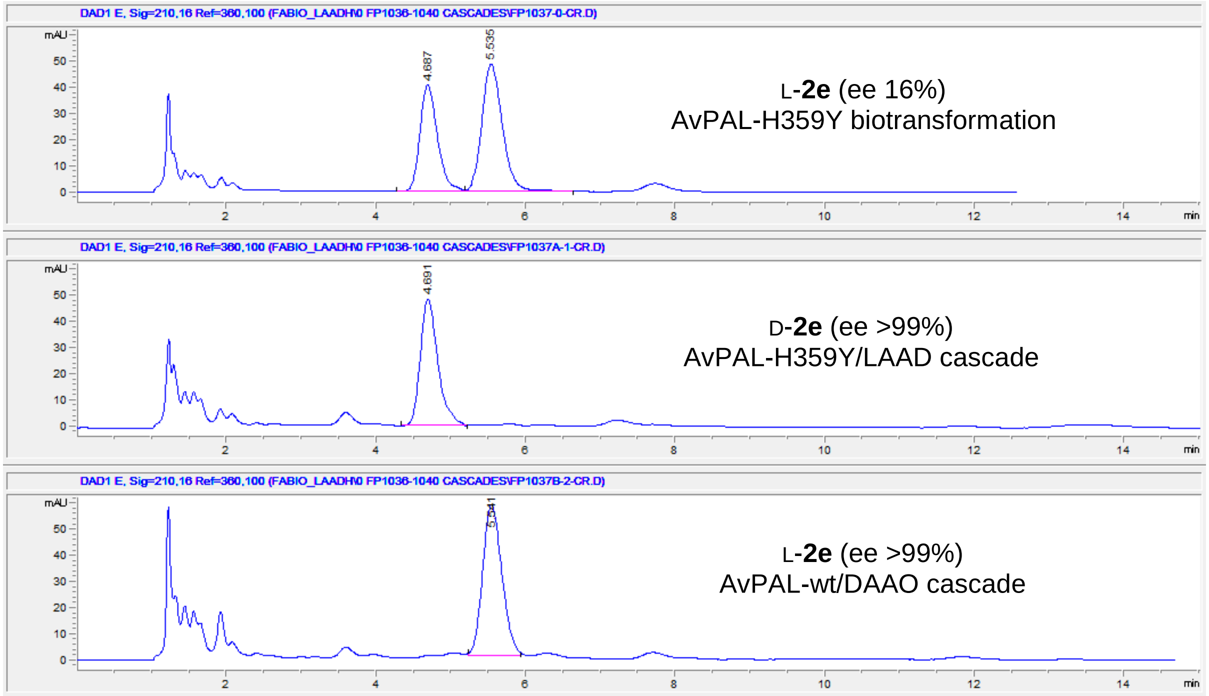
DAD1 E, Sig=210,16 Ref=360,100 (FABIO_LAADH\0 FP1036-1040 CASCADES\FP1037-0-CR.D)
L-2e (ee 16%)
AvPAL-H359Y biotransformation
DAD1 E, Sig=210,16 Ref=360,100 (FABIO_LAADH\0 FP1036-1040 CASCADES\FP1037A-1-CR.D)
D-2e (ee >99%)
AvPAL-H359Y/LAAD cascade
DAD1 E, Sig=210,16 Ref=360,100 (FABIO_LAADH\0 FP1036-1040 CASCADES\FP1037B-2-CR.D)
L-2e (ee >99%)
AvPAL-wt/DAAO cascade

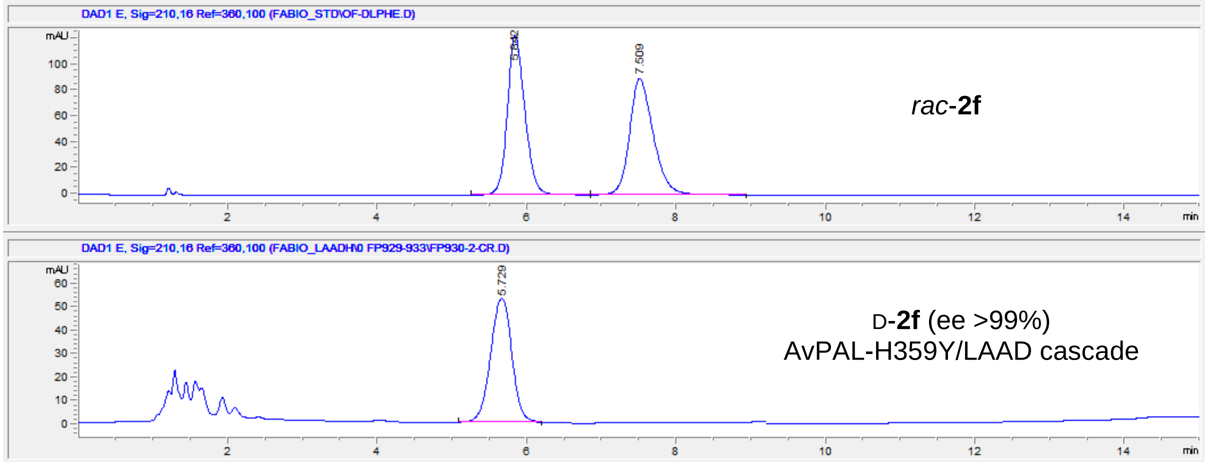
DAD1 E, Sig=210,16 Ref=360,100 (FABIO_STD\OF-DLPHE.D)
rac-2f
DAD1 E, Sig=210,16 Ref=360,100 (FABIO_LAADH\0 FP929-933\FP930-2-CR.D)
D-2f (ee >99%)
AvPAL-H359Y/LAAD cascade

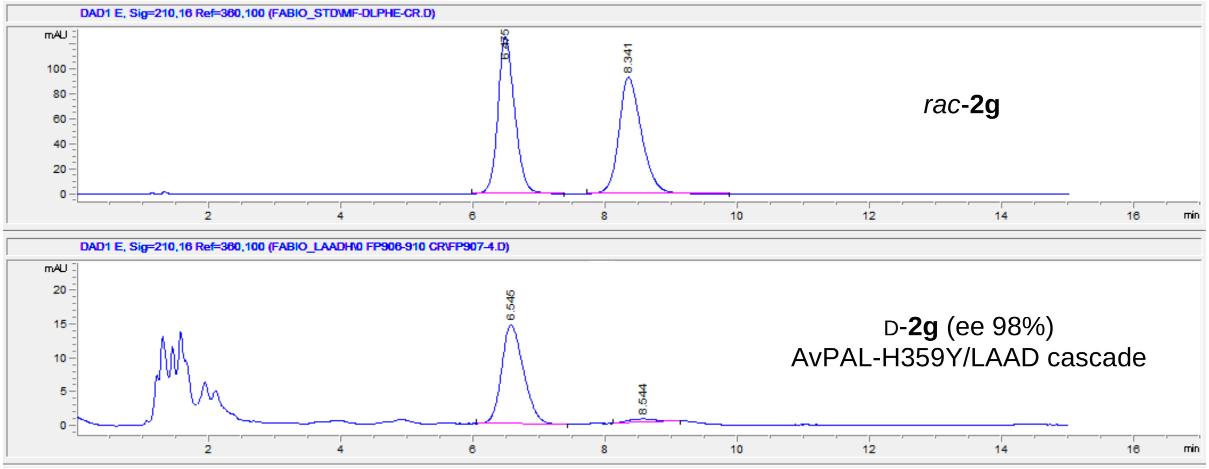
DAD1 E, Sig=210,16 Ref=360,100 (FABIO_STD\MF-DLPHE-CR.D)
rac-2g
DAD1 E, Sig=210,16 Ref=360,100 (FABIO_LAADH\0 FP906-910 CR\FP907-4.D)
D-2g (ee 98%)
AvPAL-H359Y/LAAD cascade

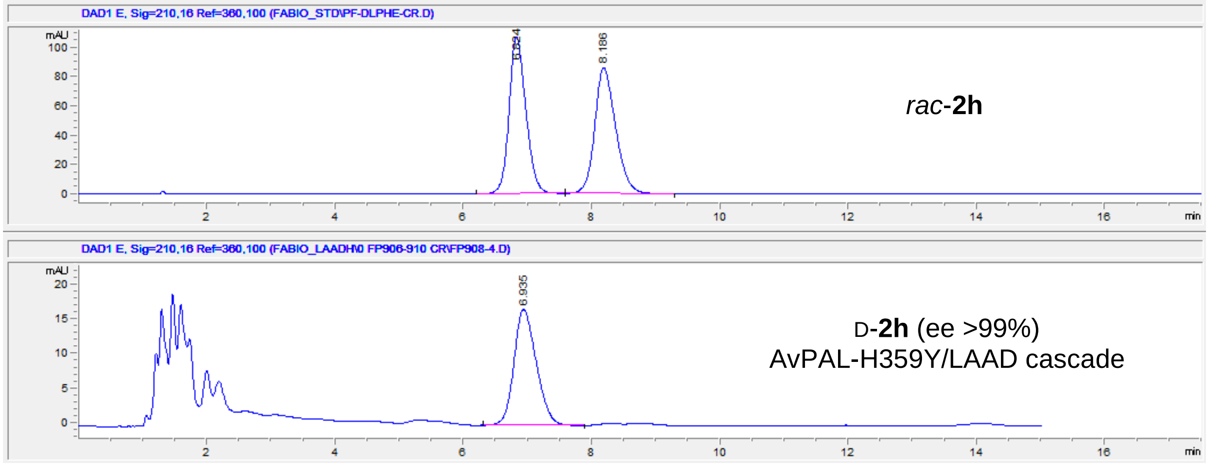
DAD1 E, Sig=210,16 Ref=360,100 (FABIO_STD\PF-DLPHE-CR.D)
8.186
*rac*-**2h**
DAD1 E, Sig=210,16 Ref=360,100 (FABIO_LAADH\0 FP906-910 CR\FP908-4.D)
6.935
D-**2h** (ee >99%)
AvPAL-H359Y/LAAD cascade

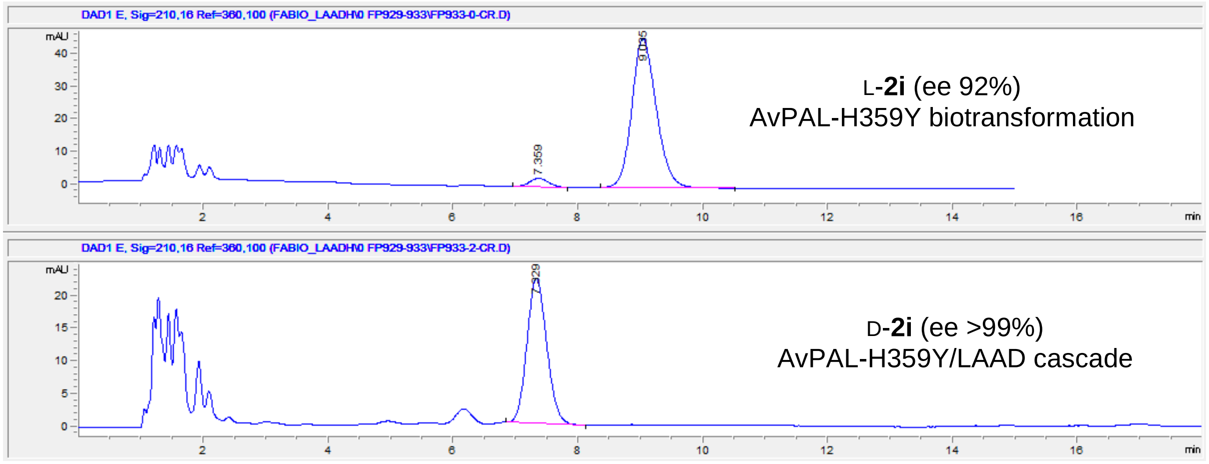
DAD1 E, Sig=210,16 Ref=360,100 (FABIO_LAADH\0 FP929-933\FP933-0-CR.D)
7.359
L-**2i** (ee 92%)
AvPAL-H359Y biotransformation
DAD1 E, Sig=210,16 Ref=360,100 (FABIO_LAADH\0 FP929-933\FP933-2-CR.D)
D-**2i** (ee >99%)
AvPAL-H359Y/LAAD cascade

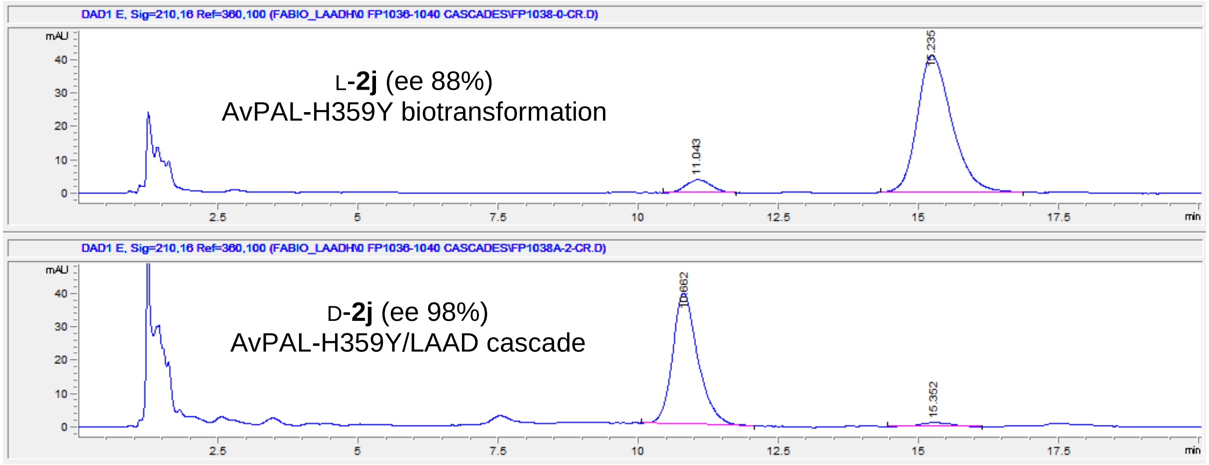
DAD1 E, Sig=210,16 Ref=360,100 (FABIO_LAADH\0 FP1036-1040 CASCADES\FP1038-0-CR.D)
L-**2j** (ee 88%)
AvPAL-H359Y biotransformation
11.043
15.235
DAD1 E, Sig=210,16 Ref=360,100 (FABIO_LAADH\0 FP1036-1040 CASCADES\FP1038A-2-CR.D)
D-**2j** (ee 98%)
AvPAL-H359Y/LAAD cascade
15.352

## $^{1}$H-NMR spectra of compounds 2a-j

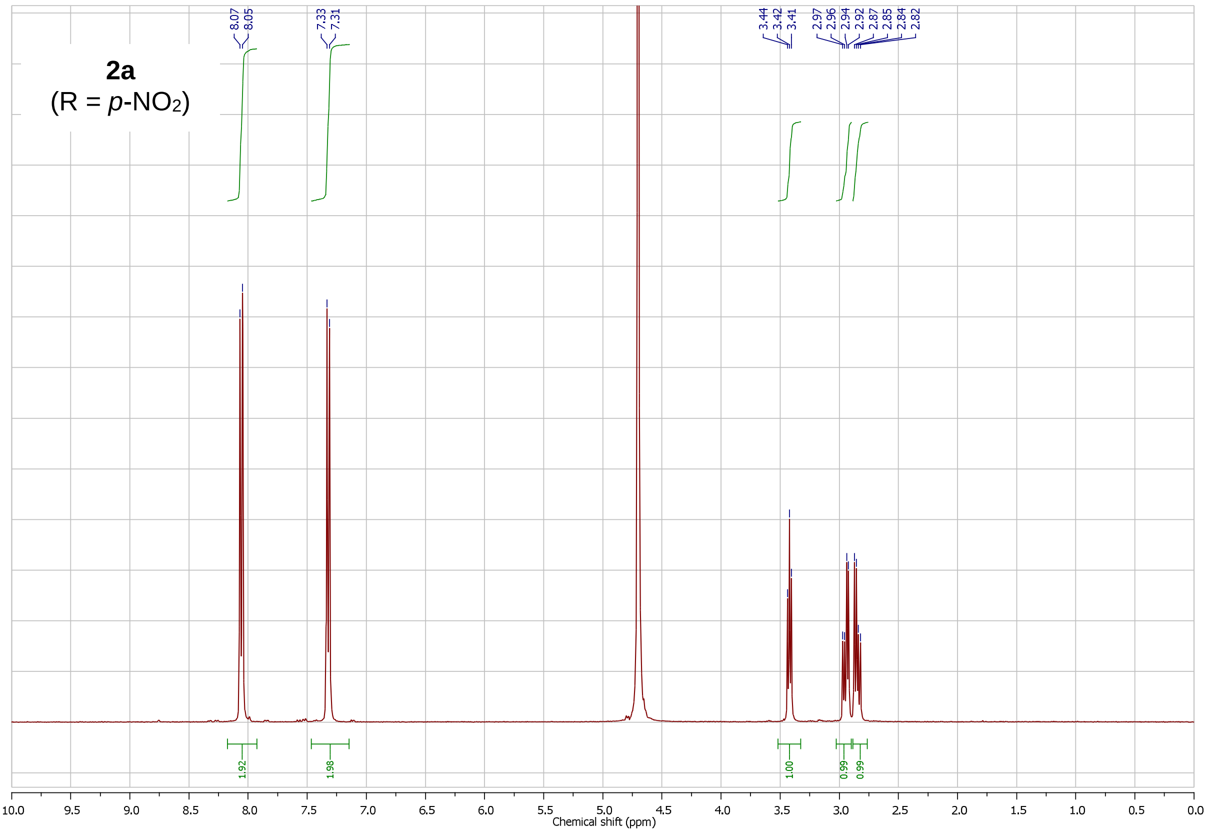


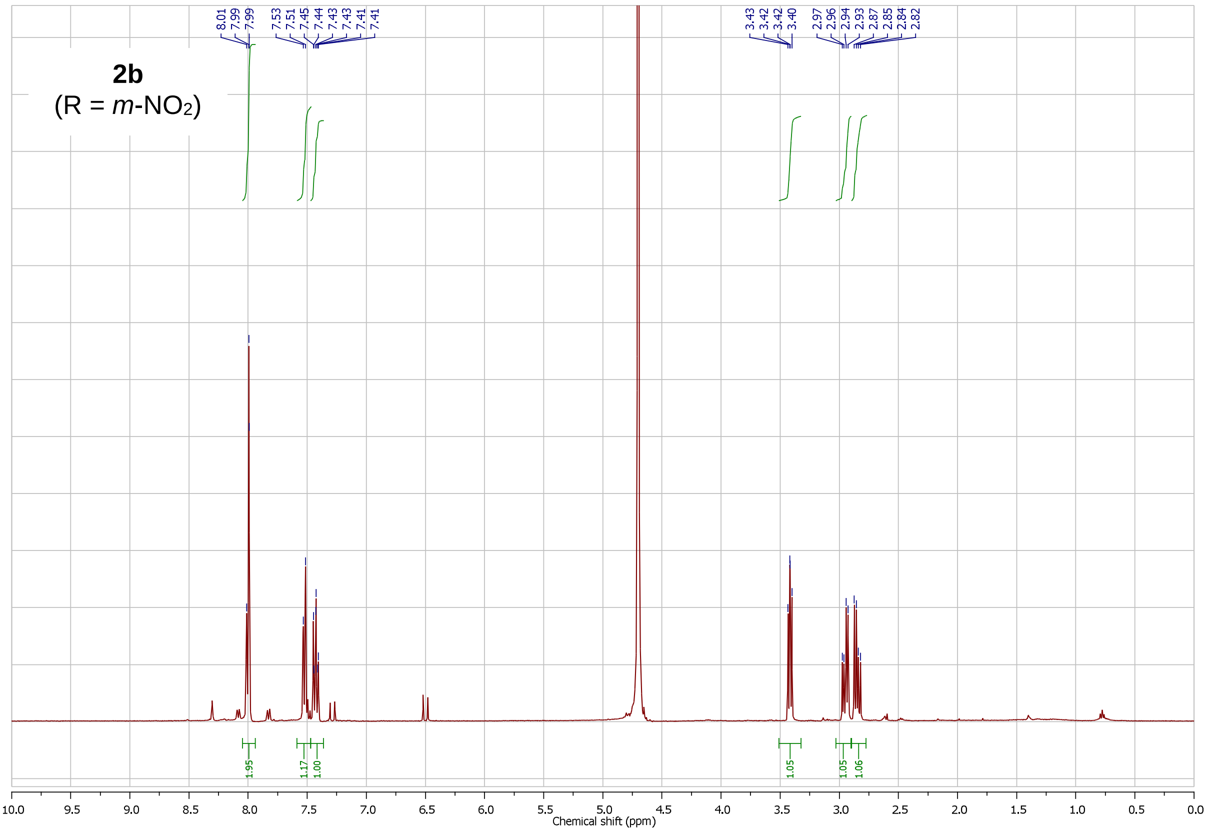

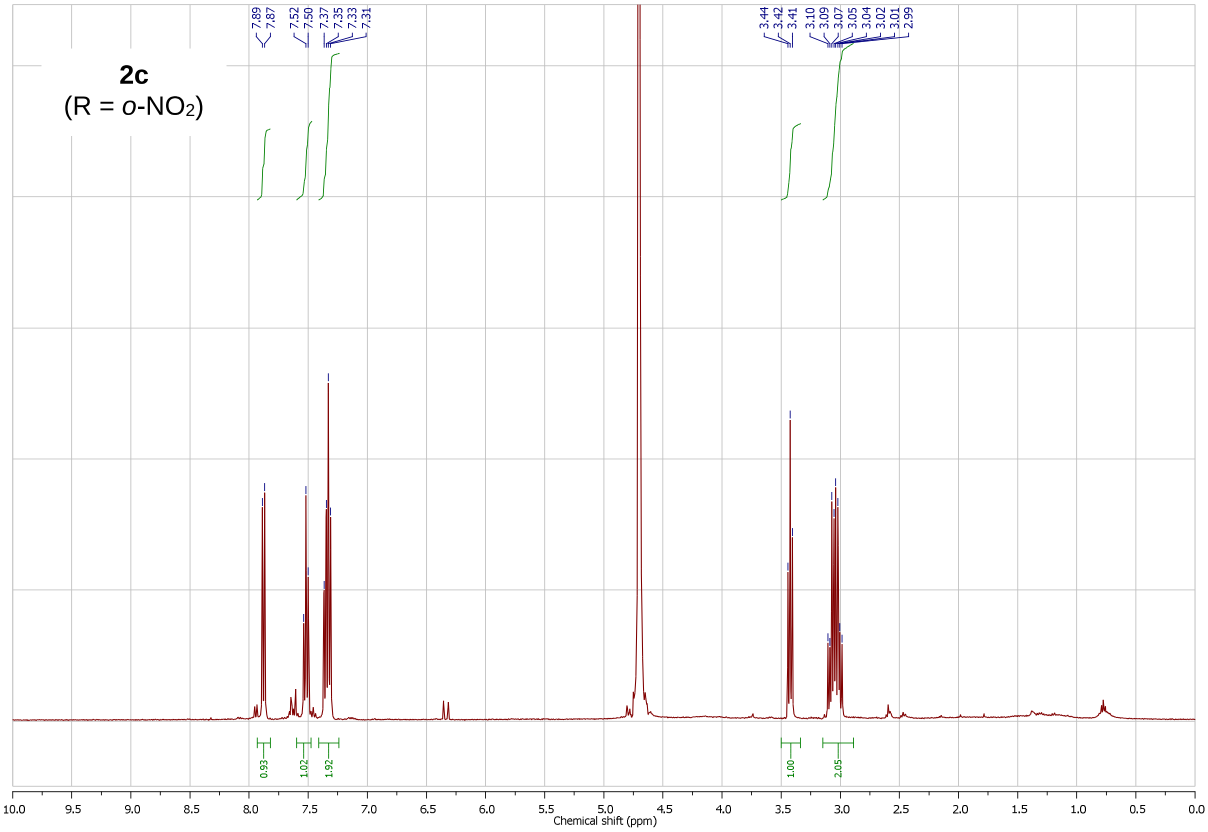
2c
(R = o-NO2)
7.89
7.87
7.52
7.50
7.37
7.35
7.33
7.31
3.44
3.42
3.41
3.10
3.09
3.07
3.05
3.04
3.02
3.01
2.99
0.93
1.02
1.92
1.00
2.05
Chemical shift (ppm)

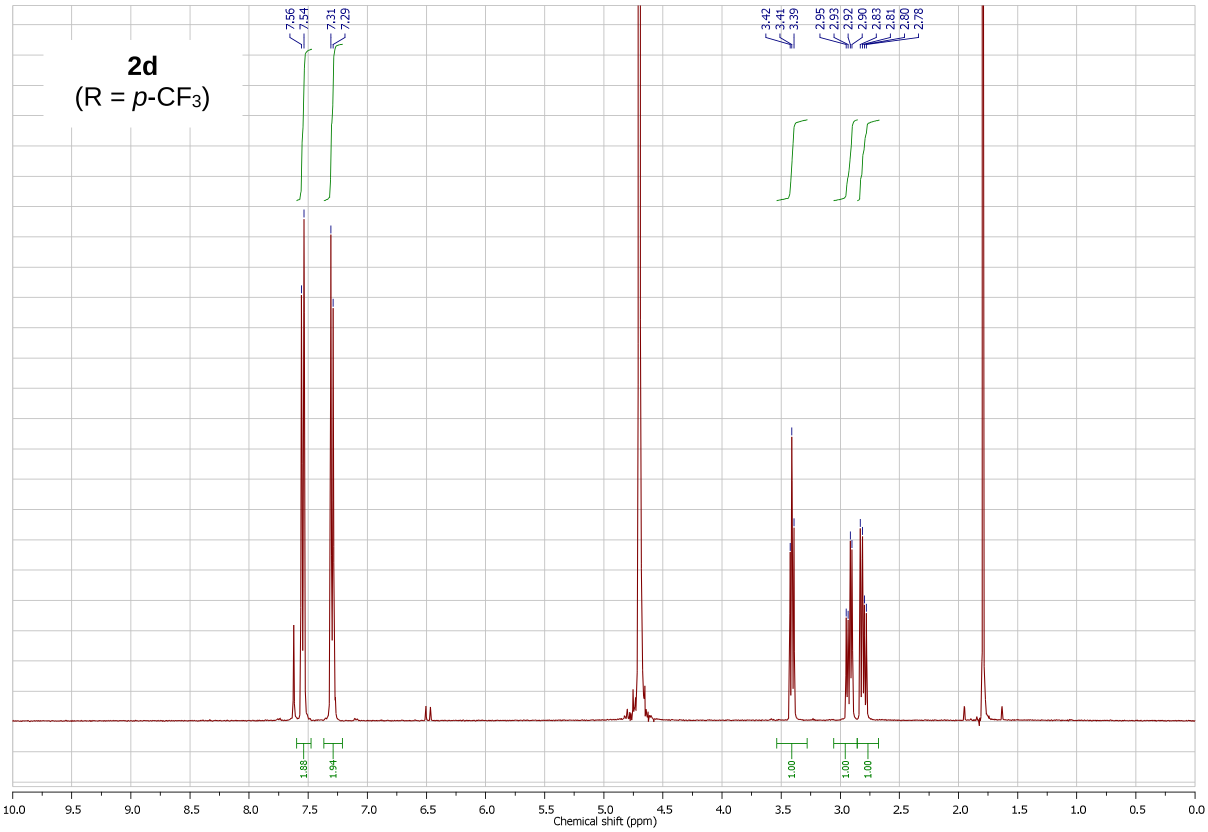
2d
(R = p-CF3)
7.56
7.54
7.31
7.29
3.42
3.41
3.39
2.95
2.93
2.92
2.90
2.83
2.81
2.80
2.78
1.88
1.94
1.00
1.00
1.00
Chemical shift (ppm)

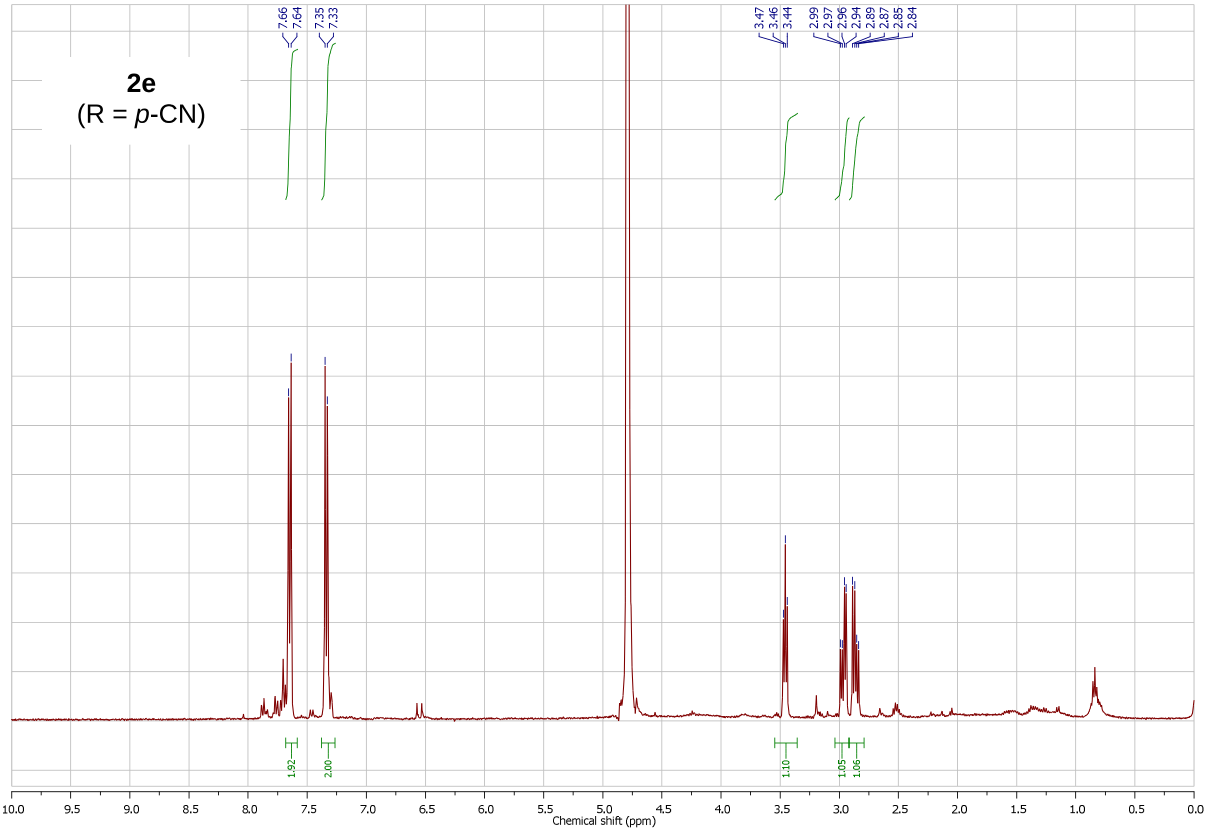
2e
(R = p-CN)
7.66
7.64
7.35
7.33
3.47
3.46
3.44
2.99
2.97
2.96
2.94
2.89
2.87
2.85
2.84
1.92
2.00
1.10
1.05
1.06
Chemical shift (ppm)

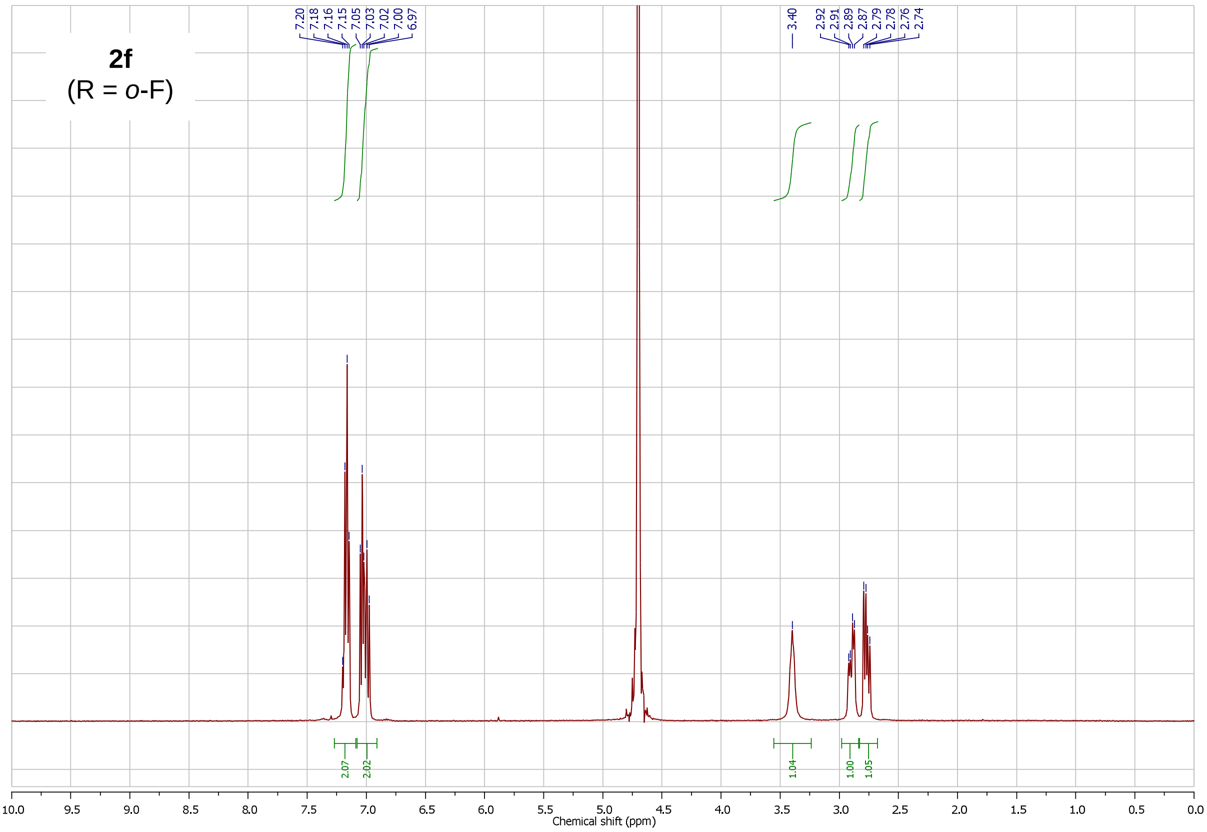
2f
(R = o-F)
7.20
7.18
7.16
7.15
7.05
7.03
7.02
7.00
6.97
3.40
2.92
2.91
2.89
2.87
2.79
2.78
2.76
2.74
2.07
2.02
1.04
1.00
1.05
Chemical shift (ppm)

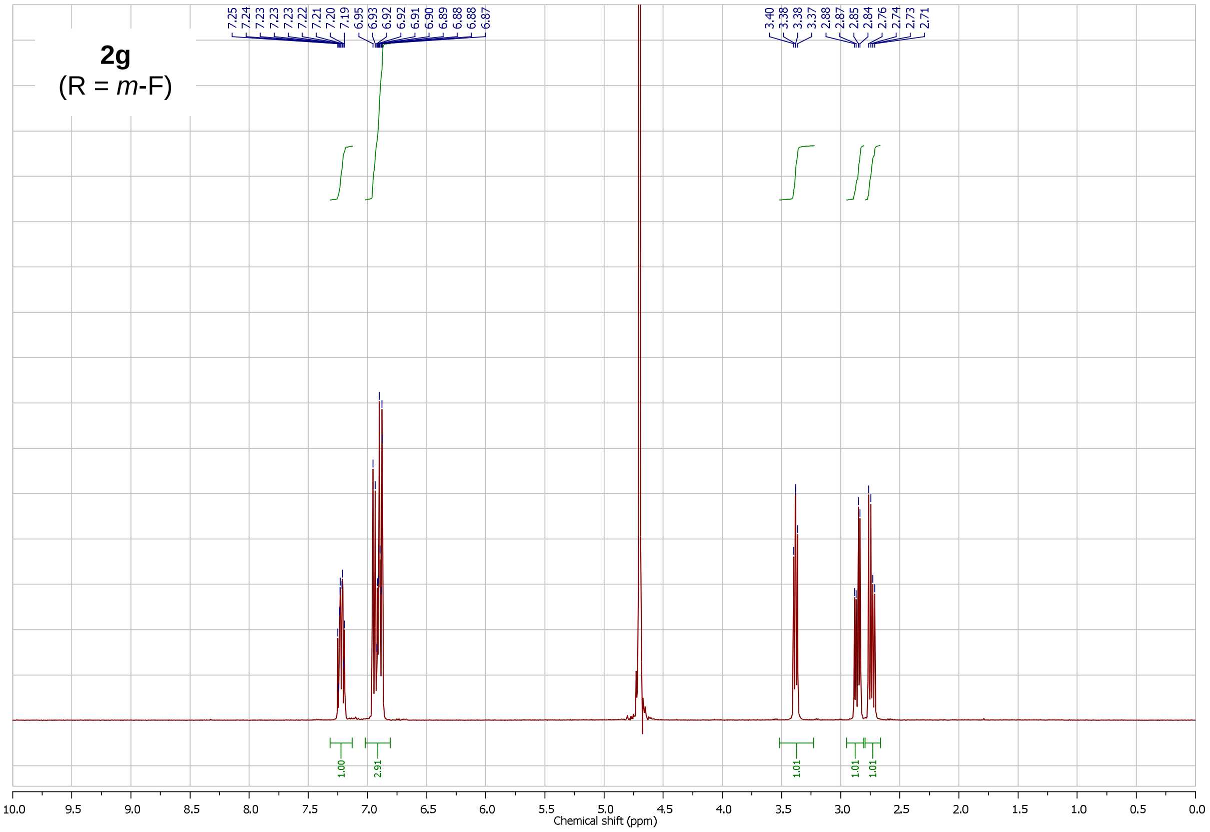

**2g**
(R = *m*-F)


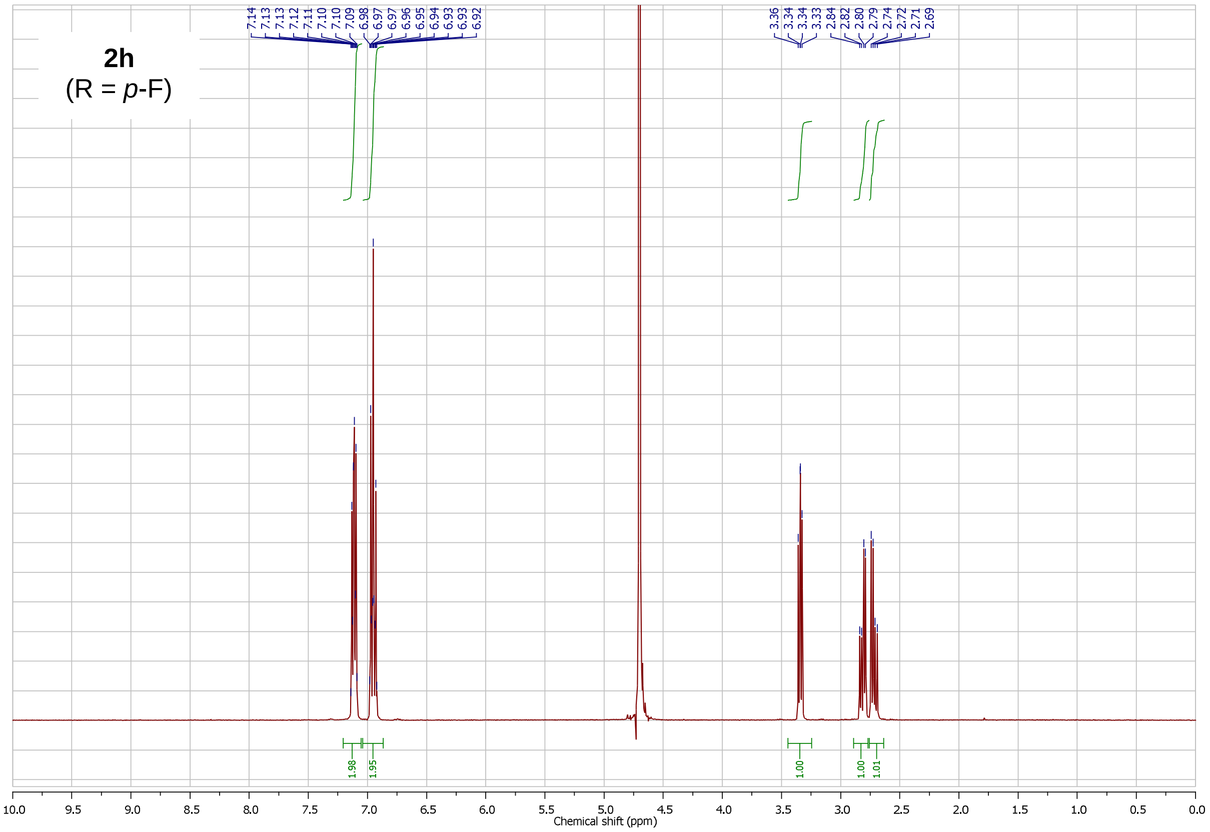

**2h**
(R = *p*-F)

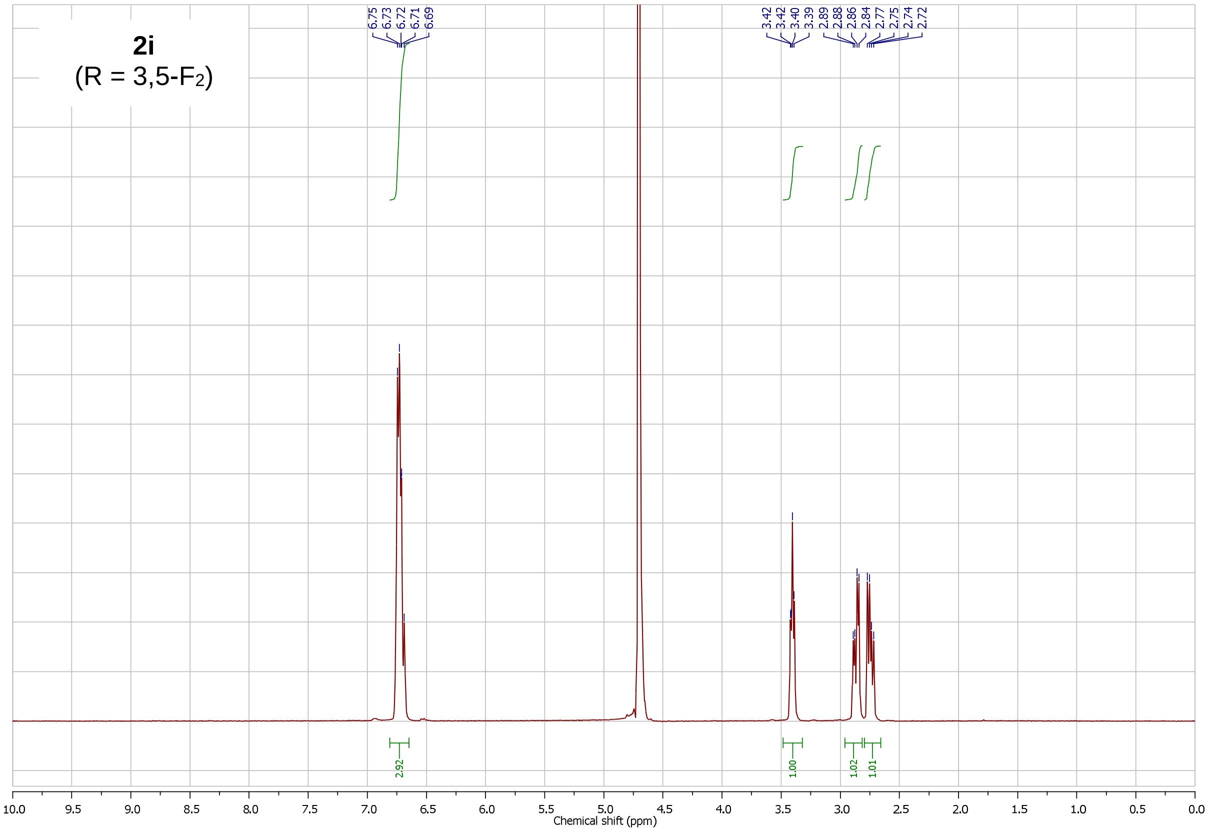
2i
(R = 3,5-F2)
6.75
6.73
6.72
6.72
6.71
6.69
3.42
3.42
3.40
3.39
2.89
2.88
2.86
2.84
2.77
2.75
2.74
2.72
2.92
1.00
1.02
1.01
10.0 9.5 9.0 8.5 8.0 7.5 7.0 6.5 6.0 5.5 5.0 4.5 4.0 3.5 3.0 2.5 2.0 1.5 1.0 0.5 0.0
Chemical shift (ppm)

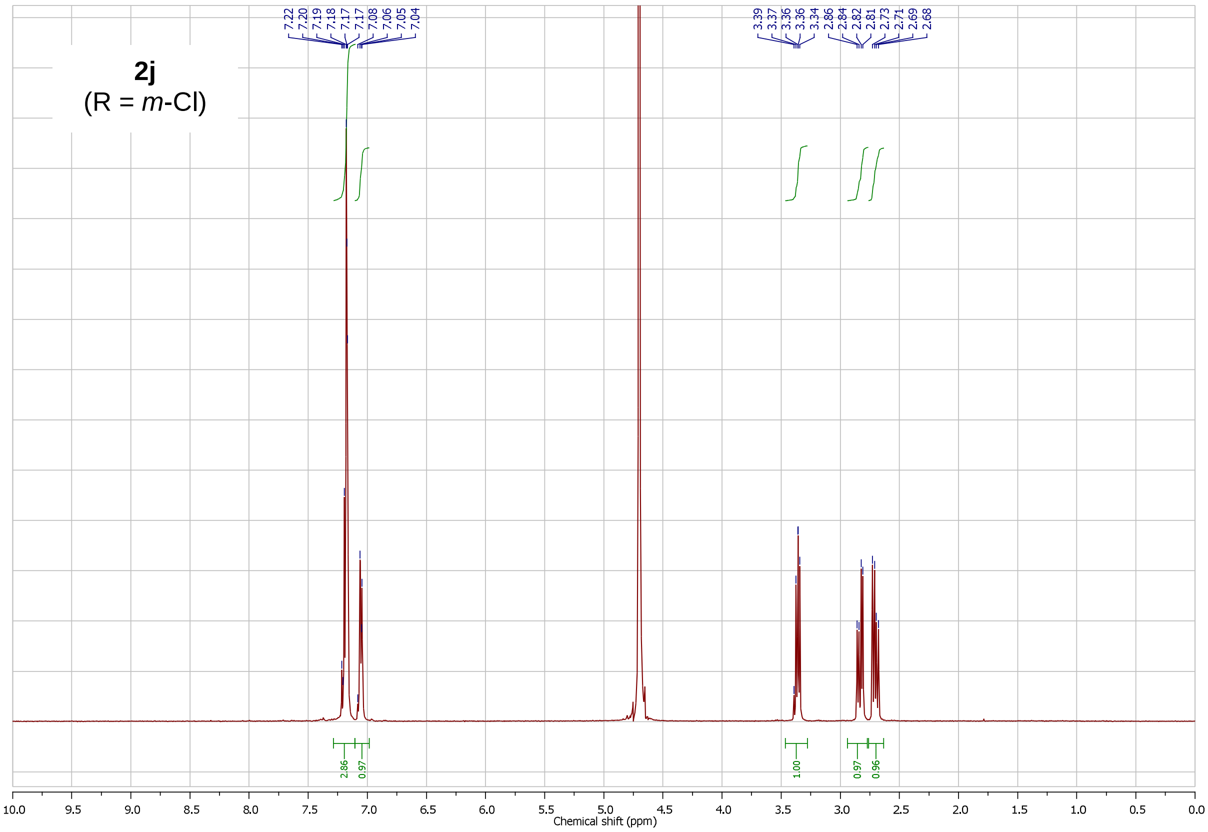
2j
(R = m-Cl)
7.22
7.20
7.19
7.18
7.17
7.17
7.08
7.06
7.05
7.04
3.39
3.37
3.36
3.36
3.34
2.86
2.84
2.82
2.81
2.73
2.71
2.69
2.68
2.86
0.97
1.00
0.97
0.96
10.0 9.5 9.0 8.5 8.0 7.5 7.0 6.5 6.0 5.5 5.0 4.5 4.0 3.5 3.0 2.5 2.0 1.5 1.0 0.5 0.0
Chemical shift (ppm)

## References


[1] S. L. Lovelock, R. C. Lloyd, N. J. Turner, *Angew. Chem. Int. Ed.* **2014**, *53*, 4652-4656.

[2] S. C. Willies, J. L. White, N. J. Turner, *Tetrahedron* **2012**, *68*, 7564-7567.

[3] F. W. Studier, *Prot. Expr. Purif.* **2005**, *41*, 207-234.
